# Hyperspectral imaging for chloroplast movement detection

**DOI:** 10.1093/jxb/erae407

**Published:** 2024-09-27

**Authors:** Paweł Hermanowicz, Justyna Łabuz

**Affiliations:** Malopolska Centre of Biotechnology, Jagiellonian University, Gronostajowa 7A, 30-387 Kraków, Poland; Malopolska Centre of Biotechnology, Jagiellonian University, Gronostajowa 7A, 30-387 Kraków, Poland; University College Dublin, Ireland

**Keywords:** *Arabidopsis thaliana*, chloroplast movements, hyperspectral imaging, leaf reflectance, *Nicotiana benthamiana*, vegetation indices

## Abstract

We employed hyperspectral imaging to detect chloroplast positioning and assess its influence on common vegetation indices. In low blue light, chloroplasts move to cell walls perpendicular to the direction of the incident light. In high blue light, chloroplasts exhibit the avoidance response, moving to cell walls parallel to the light direction. Irradiation with high light resulted in significant changes in leaf reflectance and the shape of the reflectance spectrum. Using mutants with disrupted chloroplast movements, we found that blue light-induced changes in the reflectance spectrum are mostly due to chloroplast relocations. We trained machine learning methods in the classification of leaves according to the chloroplast positioning, based on the reflectance spectra. The convolutional network showed low levels of misclassification of leaves irradiated with high light even when different species were used for training and testing, suggesting that reflectance spectra may be used to detect chloroplast avoidance in heterogeneous vegetation. We also examined the correlation between chloroplast positioning and values of indices of normalized-difference type for various combinations of wavelengths and identified an index sensitive to chloroplast positioning. We found that values of some of the vegetation indices, including those sensitive to the carotenoid levels, may be altered due to chloroplast rearrangements.

## Teaser

Computational methods enable retrieval of chloroplast positioning from hyperspectral reflectance images of leaves.

## Introduction

Plants show a remarkable ability for chloroplast relocation in changing light conditions (for a review, see Łabuz *et al.*, 2022). In low light, chloroplasts move to cell walls perpendicular to the direction of incident light. This response, called chloroplast accumulation, increases light capture for photosynthesis (Gotoh *et al.*, 2018). In high light, chloroplasts gather at cell walls parallel to the direction of incident light. Chloroplast avoidance protects the photosynthetic apparatus from excess light (Kasahara *et al.*, 2002; Sztatelman *et al.*, 2010; Davis and Hangarter, 2012) or shapes light gradients within the leaf tissue (Brugnoli and Bjorkman, 1992; Wilson and Ruban, 2020). Seed plants move chloroplasts in response to blue light; however, red light is also active in some ferns, mosses, and green algae (Suetsugu *et al.*, 2005b). In the model plant *Arabidopsis thaliana*, blue light-induced chloroplast movements are controlled by photoreceptor serine-threonine kinases called phototropins (Christie, 2007). Two phototropins, phot1 and phot2, mediate the chloroplast accumulation response (Sakai *et al.*, 2001). Sustained chloroplast avoidance can be elicited only by phot2 (Jarillo *et al.*, 2001; Kagawa *et al.*, 2001). Thus, no substantial directional chloroplast movements are observed in the *phot1phot2* double phototropin mutant. The *phot1* mutant is less sensitive to very low light irradiances than wild-type plants, but it shows both accumulation and avoidance response (Luesse *et al.*, 2010). In the *phot2* mutant, light elicits chloroplast accumulation regardless of irradiance, though it is preceded by a weak, transient avoidance response in high light (Luesse *et al.*, 2010; Łabuz *et al.*, 2015). Chloroplast avoidance is observed in both low and high light in *jac1*, a signaling mutant lacking JAC1, the J-domain protein required for chloroplast accumulation response 1 (Suetsugu et al., 2005a).

The first microscopic chloroplast movement observations were made by Senn (1908), revealing a plethora of different arrangements within plant cells (Kataoka, 2015). Microscopic observations still play a pivotal role in assessing chloroplast positions within leaf cells, but they are laborious (Kagawa and Wada, 2000) and provide information for individual cells. Interpretation of microscopic observations requires care due to the steep fluence rate gradient within the mesophyll tissue. In some species, the effects of chloroplast movements on the optical properties of the leaves are so pronounced that they may be observed with the naked eye. Whereas chloroplast avoidance results in paler leaf blades, chloroplast accumulation causes the darkening of leaves. This effect is the basis for the so-called slit assay (Kagawa and Wada, 2000), and is even utilized by artists (Dolinsky and Hangarter, 2013). Changes in leaf transmittance correspond to averaged chloroplast positions within the mesophyll and are used to assess chloroplast movements quantitatively. The development of photometric devices for measuring blue light-induced changes in leaf transmittance offered a convenient approach, however they were limited in throughput and restricted mainly to laboratory use (Shibata, 1958; Yorinao and Kazuo, 1974; Walczak and Gabrys, 1980). Leaf reflectance has also been used to monitor chloroplast movements (Park *et al.*, 1996; Gorton *et al.*, 1999). A method using measurements of red light reflected from the leaf surface was developed to monitor movements during the growth of *A. thaliana* in a growth chamber (Dutta *et al.*, 2015). Analysis of leaf optical properties for several species indicated an important role for the cell shape in the ability of chloroplast to move and subsequently alter the leaf absorptance and scattering (Davis *et al.*, 2011). A detailed study of leaf reflectance has been performed using *Nicotiana tabacum.* Chloroplast positioning resulted in the highest changes in the leaf reflectance spectrum in the region between 500 nm and 650 nm. The changes in leaf reflectance were ~2% in the blue and red regions and ~5% in the green region of the light spectrum (Baránková *et al.*, 2016). The works of Davis *et al.* (2011) and Baránková *et al.* (2016) used an integrating sphere to measure changes in diffuse reflectance. While this approach yields invaluable data on the effects of chloroplast movements on the optical properties of the leaves, it shares the limitations of the transmittance-based method in its low throughput and requirement for direct contact with the leaf.

The aims of our work were 2-fold—to establish a system for high-throughput chloroplast relocation monitoring using spectra of white light reflected from leaves and to examine whether changes in chloroplast positioning can confound the assessment of leaf traits from commonly used vegetation indices. We have used multiple mutant lines that exhibit disrupted chloroplast movements to distinguish between changes in leaf spectra that can be ascribed to chloroplast relocations from changes due to other light-induced processes. To acquire reflectance spectra and examine their spatial variability within the leaf, we have used hyperspectral imaging, which is a high-throughput, contactless method.

Vegetation indices are a convenient approach to reduce the possibly redundant information in spectra, focusing on the most relevant wavelengths or bands. Hyperspectral or narrow-band vegetation indices are single values calculated using a combination of wavelengths, which are empirically shown to correlate with the traits of interest. Vegetation indices were developed to examine plant or canopy structure (e.g. fractional cover, green biomass, leaf area index, and senesced biomass), as well as biochemical (e.g. the content of water, nitrogen, lignin, cellulose, and pigments: chlorophyll, carotenoids, and anthocyanins) and physiological traits (e.g. stress, changes in xanthophyll cycle pigments, fluorescence, and leaf moisture) (Thenkabail *et al.*, 2018). In particular, a large group of vegetation indices based on the visible or near infrared (NIR) reflectance (formulae in Supplementary Table S1) is used to assess the chlorophyll content, consisting of the classical normalized difference vegetation index (NDVI; Rouse *et al.*, 1974), but also the simple ratio index (SR; Rouse *et al.*, 1974), enhanced vegetation index (EVI; Huete *et al.*, 1997), atmospherically resistant vegetation index (ARVI; Kaufman and Tanre, 1995), red edge NDVI (RENDVI; Gitelson and Merzlyak, 1994), modified red edge simple ratio index (mRESR; Sims and Gamon, 2002), and a sum green index (SG; Behmann *et al.*, 2014). Vogelmann red edge index 1 (VOG1), VOG2, and VOG3 link the chlorophyll and water contents (Vogelmann *et al.*, 1993). The photochemical reflectance index (PRI; Gamon *et al.*, 1992), structure-insensitive pigment index (SIPI; Penuelas *et al.*, 1995), carotenoid reflectance index 1 (CAR1), and CAR2 (Gitelson *et al.*, 2002) represent carotenoid proportions, while the red–green ratio index RGRI; Gamon and Surfus, 1999) represents the anthocyanin–chlorophyll ratio. The modified red edge NDVI (mRENDEVI; Sims and Gamon, 2002) and plant senescence reflectance index (PSRI; Merzlyak *et al.*, 1999) are used for stress and senescence estimation. The equations specifying vegetation indices are based on the visible and NIR range of the solar spectrum. In the visible and NIR range, several bands were found to be particularly useful for remote sensing, including 660–690 nm (red absorption maxima), 900–925 nm (NIR reflection peak), 700–720 nm (a portion of red edge), and 540–555 nm (green reflectance maxima), a 490 nm centered band (rapid change in slope of the spectra), 520 nm and 575 nm bands (the most rapid positive or negative change in reflectance), a 845 nm centered band (the NIR shoulder), and a 975 nm centered band (biomass/moisture sensitive) (Thenkabail *et al.*, 2002). The estimates of plant traits based on vegetation indices may be biased due to the effects of other, non-specific physiological responses on the leaf reflectance, such as senescence (Idso *et al.*, 1980), as well as changes in the chlorophyll and carotenoid content (Tucker, 1977). The values of vegetation indices may also be altered by topography (Chen *et al.*, 2020) or snow cover (Wang *et al.*, 2023). In this work, we have investigated the impact of chloroplast positioning on commonly used vegetation indices and discussed the magnitude of their changes caused by chloroplast movements compared with the scale of changes due to the variation of other traits, reported in the literature. We have analyzed the accuracy of vegetation indices for the detection of chloroplast positioning and identified an index of the normalized difference type, tentatively referred to as a chloroplast movement index (CMI), that correlates well with the chloroplast arrangement.

To examine whether a more accurate classification of leaves according to chloroplast arrangement can be obtained using whole spectra of reflectance in the visible range, we have examined the performance of three supervised machine learning procedures in the task of classifying leaves according to the chloroplast positioning based on the reflectance spectra. We employed the support vector machine and two types of neural feed-forward network architectures, namely the multilayer perceptron and convolutional networks. A mathematically rigorous treatment of both of these types of neural networks can be found in Caterini and Chang (2018) and Berner *et al.* (2023). The multilayer perceptron (Pal and Mitra, 1992) is a classical architecture, consisting of a variable number of densely connected layers of neurons. It has been used already in the early works applying machine learning for the analysis of reflectance spectra and remotely sensed data, as reviewed in Mas and Flores (2008). In particular, it has been successfully applied to the detection of fungal infections from leaf spectra (Moshou *et al.*, 2004) and to estimate carotenoid content through inversion of the PROSPECT model (Hao *et al.*, 2023). In convolutional neural networks, densely connected layers resembling the multilayer perceptron are preceded by additional layers that convolve their input with an arbitrary number of kernels. The benefits of convolutional networks include their good generalization (Alzubaidi *et al.*, 2021) so that the network performance is usually not substantially degraded when applied to an unseen dataset. They have recently become a popular tool for analysis of spectral data. Their applications include the prediction of the concentration of photosynthetic pigment through the inversion of leaf optical models (Annala *et al.*, 2020; Shi *et al.*, 2022) as well as the estimation of nitrogen (Pullanagari *et al.*, 2021) and water content (Zhang *et al.*, 2023) from the leaf reflectance spectra. However, an architecture based on the multilayer perceptron was found to be superior to a convolutional network for the prediction of photosynthetic performance in Japanese beech trees based on leaf reflectance (Song and Wang, 2021).

## Materials and methods

### Plants and growth conditions

Seeds of *Nicotiana benthamiana*, *Arabidopsis thaliana* wild-type Col-0, *phot1* (SALK_088841) (Lehmann *et al.*, 2011), the *phot2* (*npl1-1*) mutant (Jarillo *et al.*, 2001), *phot1phot2* (SALK_088841 crossed with *npl1-1*) (Hermanowicz *et al.*, 2019), and *jac1-3* (WiscDsLox457-460P9) (Hermanowicz *et al.*, 2019) were sown in Jiffy-7 pots (Jiffy Products International AS) and vernalized at 4 °C for 2 d. Arabidopsis mutants were always grown together with wild-type plants to avoid a systematic bias. All plants were transferred to a growth chamber (Sanyo MLR 350H) at 23 °C, 80% relative humidity, with a photoperiod of 10 h light and 14 h darkness. The irradiance at the plane of leaf rosettes was 70 μmol m^–2^ s^–1^. Light was supplied by fluorescent lamps (FL40SS.W/37, Sanyo, Japan). After 4 weeks, *N. benthamiana* were transferred to pots containing soil and moved to a walk-in growth chamber with a photoperiod of 10 h light and 14 h darkness, equipped with white LEDs supplying irradiance of ~100 μmol m^–2^ s^–1^. Four-week-old Arabidopsis and 6-week-old *N. benthamiana* plants were used for the experiment. Mature, fully developed leaves of Arabidopsis and *Nicotiana* with no signs of senescence were chosen for experiments. Plants were dark-adapted for at least 16 h for all kinds of measurements of chloroplast movements. For microscopic observations, detached leaves were either kept in darkness or irradiated with blue light of 1.6 µmol m^–2^ s^–1^ or 120 µmol m^–2^ s^–1^ for 1 h (EPILED, 1 W, 460 nm). For hyperspectral imaging, half of the leaf was covered with aluminum foil and the other half was irradiated with the same light irradiance as for confocal microscopy. The division line between the darkened and irradiated leaf parts ran along the main vein. Total chlorophyll content per unit of leaf area was measured using a Leaf State Analyzer (Walz, Germany) in plants growing in the same conditions as those used for the experiment with the hyperspectral camera. For *N. benthamiana*, chlorophyll content was examined in the top, middle, and bottom parts of the blade of dark-adapted plants, separately on the left and right sides of the blade. Chlorophyll content was also measured in wild-type and *phot1phot2* mutant Arabidopsis leaves.

### Microscopic observations

Microscopic observations were performed with the Axio Observer.Z1 inverted microscope (Carl Zeiss, Jena, Germany) and the LSM 880 confocal module. The long-distance LCI Apochromat ×25, NA 0.8, objective was used with glycerol immersion. Chloroplast positioning was observed in the palisade parenchyma of wild-type *N. benthamiana* and *A. thaliana* leaves, as well as *phot1*, *phot2*, *phot1phot2*, and *jac1* mutants. The 633 nm He–Ne laser was used to excite chlorophyll, and emission in the range of 661–721 nm was recorded. Stacks were collected on the upper surface of the leaves. Projection images were calculated from slices corresponding to the first ~90 μm of stacks, starting from the upper surface of the epidermis. This range included the epidermis and upper parts of palisade cells.

### Photometric method

Leaf transmittance changes due to chloroplast movements were assessed with the photometric method (Gabryś *et al.*, 2017), using a custom-built photometric set up. Chloroplast responses were induced with blue (peak at 455 nm, M455L4 LED, Thorlabs) actinic light of 1.6 µmol m^−2^ s^–1^ or 120 µmol m^−2^ s^–1^. The red measuring beam was produced by a 660 nm LED (M660L4, Thorlabs) and modulated at 1033 Hz. The beams were collimated and combined with a dichroic mirror (DMLP550, Thorlabs). They were then directed toward a detached leaf, mounted in front of a port of an integrating sphere (IS200-4, Thorlabs). The signal was detected with a photodiode detector (DET100A2, Thorlabs) mounted at another port of the sphere. Leaf transmittance curves were processed with a custom-written Mathematica (Wolfram Research) script. Irradiance was measured at the sample plane with the LI-190R sensor (LI-COR) and Keithley 6485 picoammeter.

### Hyperspectral imaging

Immediately after blue irradiation, the aluminum foil was removed from the covered leaf half, and hyperspectral images of whole leaves were collected using a Headwall Nano hyperspectral camera (Nano-Hyperspec VNIR 400-1000 nm), equipped with a 12 mm objective (Cinegon F/1.4/12-0906, C-Mount, Schneider Kreuznach, 400–1000 nm). The camera was mounted on a rotating platform (Sevenoak SK-ebh01 Pro), 30 cm from the table. The camera recorded light reflected from the leaf in the range of 400–1000 nm. A halogen lamp (Hedler), located 58 cm from the table, was used as a white light source delivering irradiance of 280 µmol m^−2^ s^–1^ in the leaf plane in the range of 400–700 nm, as measured with the LI-190R sensor (LI-COR). The angle of incidence of light with respect to the leaf normal was ~10°. The camera was mounted on a rotary base and the angle of observation varied depending on the point of the leaf blade, but was smaller in magnitude than 10°. The directions of irradiation and observation, as well as the leaf normal, were contained in the same plane. All images were taken on a black velvet background. The time required to record a single image was 25–30 s. White, diffusely reflecting PTFE disks (SM05CP2C, Thorlabs) were used as reflectance standards. The extraction of spectra and calculation of the mean difference in reflectance spectra between irradiated and darkened halves were performed using a custom script written in Mathematica 13.3 (Wolfram Research). The steps of the procedure used to calculate the relative reflectance of the leaves are shown in Supplementary Fig. S1.

### Machine learning procedures for leaf classification

The performances of three types of machine learning procedures, the support vector machine, the multilayer perceptron, and the convolutional network, in the classification of leaves based on reflectance spectra were examined using Mathematica 13.3 (Wolfram Research). Spectra were normalized, truncated to the 400–750 nm range, and divided into three classes, corresponding to dark-adapted, low-light-, and high-light-irradiated leaves. As a data augmentation procedure, spectra were extracted from square blocks (60×60 pixels), randomly selected within the leaf area. Training and validation were performed using spectra recorded on *N. benthamiana* leaves. To assess the generalization capability of the trained classifiers, final testing was performed on spectra recorded from *N. benthamiana*, as well as on spectra from *A. thaliana*. The *N. benthamiana* dataset was first split into the test set (25%) and the set used for building classifiers (75%). The latter was further split into the training set and the validation (held-out) set, using balanced 5-fold cross-validation. Cross-validation was repeated 10 times, with different random partitioning of the dataset into the folds. The performance of the trained classifiers on the validation set guided the selection of model hyperparameters. For the support vector machine, five kernel types were examined (linear, polynomial of degrees 3 and 4, sigmoid, and radial Gaussian function). Multilayer perceptron architectures with three or fewer densely connected layers were examined, with up to 30 neurons per layer and a decreasing number of neurons in the input–output direction. Each layer was followed by a batch normalization layer and a rectified linear unit (ReLU). For the convolution networks, we examined several combinations of the number of convolution layers (1–4), the width of the convolution and maximum pooling kernels, the number of feature maps, as well as the number of densely connected layers. Successive convolutional layers had decreasing kernel width and increasing number of feature maps, which is a common approach. ReLUs were always used for activation functions, and batch normalization layers were inserted after each densely connected layer to regularize the trained network. The final layer of neurons in the multilayer perceptron and convolutional networks was connected to a softmax layer. As the modeled response variable was categorical, with three classes, the training consisted of minimizing the negative log-likelihood of the multinomial distribution, corresponding to the cross-entropy function. The training was carried out in 2000 epochs, using the Adam optimizer, with a batch size of 64.

### Statistical analysis

Statistical analysis was performed using the R software (R Core Team, 2019). Response variables (differences between values of vegetative indices for irradiated and darkened leaf sides) were modeled as linearly dependent on either one fixed factor (irradiation conditions, for *N. benthamiana*) or two fixed factors with an interaction term (irradiation conditions and the plant line, for *A. thaliana*). The linear models were fitted using the *gls* command of the *nlme* package (Pinheiro and Bates, 2000; Pinheiro *et al.*, 2023). The fitted models allowed for the variance to differ between groups. For Arabidopsis, the statistical significance of differences in means between groups was examined using Tukey’s method. Calculations were performed combining the *emmeans* (Lenth, 2023) and the *multcomp* package (*cld* method) (Hothorn *et al.*, 2008). As there were only two groups for *N. benthamiana* (corresponding to high and low light), controlling for the familywise error rate, achieved by Tukey’s test, was not necessary. In this case, the differences between means were calculated using the *t-*test with the Welch correction for unequal variances.

## Results

### Hyperspectral imaging of chloroplast movements in *Nicotiana benthamiana*

**Fig. 1.**
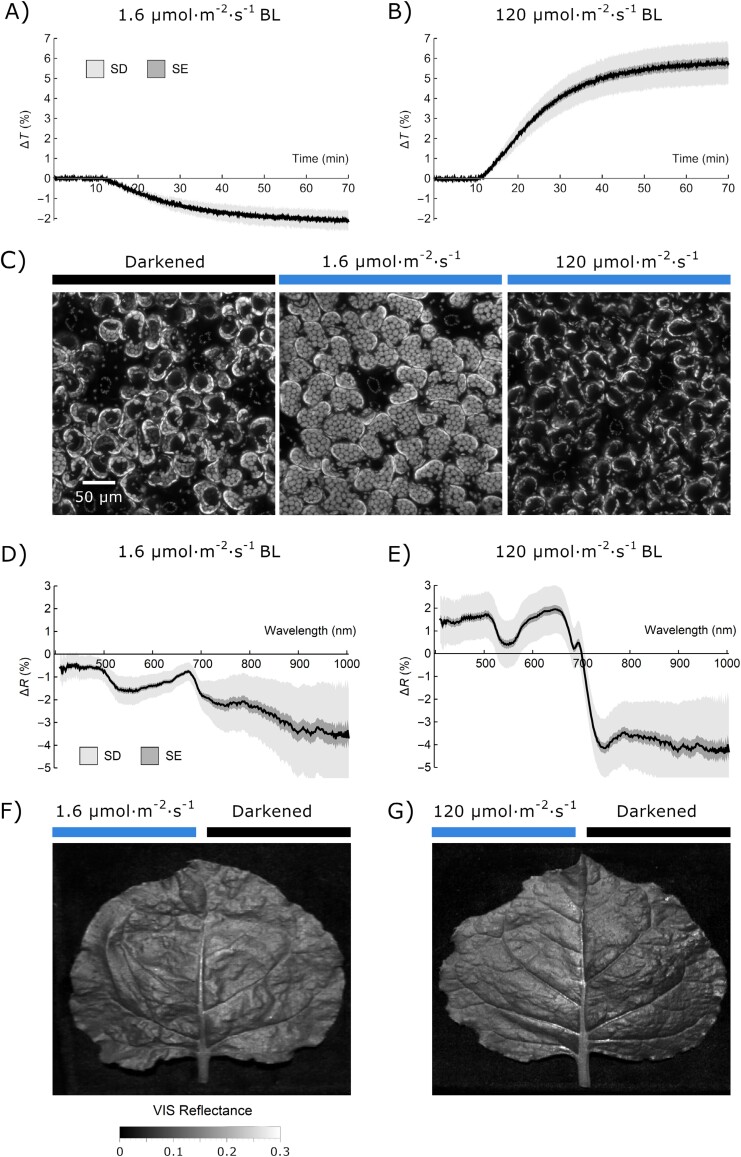
Chloroplast movements in *Nicotiana benthamiana* leaves. The blue light-induced movements were assessed with (A, B) transmittance measurements, (C) microscopic observations, and (D–G) leaf reflectance measurements. (A, B) Changes in leaf transmittance at 660 nm, induced by blue light of (A) 1.6 µmol m^−2^ s^−1^ or (B) 120 µmol m^−2^ s^−1^. Each curve represents the mean of 10 measurements. The SD is shown in light gray, and the SE is in dark gray. A decrease in leaf transmittance corresponds to chloroplast accumulation and an increase to chloroplast avoidance. (C) Chloroplast arrangements in palisade cells of *N. benthamiana*. Leaves detached from 6-week-old dark-adapted plants were either kept in darkness (mock irradiation) or irradiated for 1 h with blue light of 1.6 µmol m^−2^ s^−1^ or 120 µmol m^−2^ s^−1^. Chloroplast arrangements were imaged with a laser-scanning confocal microscope, using chlorophyll autofluorescence. Maximum intensity projections were calculated from *Z*-stacks, recorded for ~90 μm, starting from the leaf’s upper surface. (D–G) Effect of blue light irradiation on the reflectance spectrum of *N. benthamiana* leaves. Detached leaves were irradiated with either 1.6 µmol m^−2^ s^−1^ or 120 µmol m^−2^ s^−1^ continuous blue light (455 nm) for 1 h, with half of the blade covered with aluminum foil. (D, E) The mean difference in reflectance spectra between irradiated and darkened leaf halves calculated from hyperspectral images. The sample size was 23 for low- and 31 leaves for high-light irradiation. The SD is shown in light gray, and the SE is in dark gray. (F, G) Reflectance images of tobacco leaves, calculated as an average of the hyperspectral images in the visible (400–700 nm) range.

To assess the accuracy of hyperspectral imaging for detecting chloroplast movements, we chose a plant with leaf blades of a considerable size, *N. benthamiana*, which exhibited substantial chloroplast movements (Davis *et al.*, 2011). As we divided the leaf along the main vein, we examined the total chlorophyll content per unit of leaf area in six places on the blade, lying symmetrically on both sides of the leaf. The chlorophyll content was greatest in the top part of the blade, though the difference between blade parts was not statistically significant (Supplementary Fig. S2A, B). Blue light of 1.6 μmol m^–2^ s^–1^ induced a decrease in leaf transmittance corresponding to chloroplast accumulation, whereas blue light of 120 μmol m^–2^ s^–1^ increased leaf transmittance in line with the chloroplast avoidance response (Fig. 1A, B). The changes in chloroplast positions due to blue light irradiation observed under the microscope (Fig. 1C) were visible in the hyperspectral images of partly irradiated leaves (Fig. 1F, G). The leaf part irradiated with blue light of 1.6 μmol m^–2^ s^–1^ for 1 h was darker than the part kept simultaneously in darkness by covering it with aluminum foil. The leaf part irradiated with blue light of 120 μmol m^–2^ s^–1^ for 1 h appeared paler than the part kept under the aluminum foil (Fig. 1F, G). The mean reflectance of dark-adapted leaves was ~11% in visible light (400–700 nm). The average difference of reflectance in this range between leaves with chloroplasts in avoidance and accumulation positions was ~2.3% (Fig. 1D, E). Differential leaf reflectance curves were calculated from the spectra recorded for non-irradiated and blue light-irradiated leaf parts (Supplementary Fig. S3; Fig. 1D, E). After irradiation with 1.6 μmol m^–2^ s^–1^, a decrease in differential leaf reflectance curves induced by blue light was observed in the green–yellow region (500–600 nm). Irradiation with blue light of 120 μmol m^–2^ s^–1^ resulted in a characteristic ‘saddle’ in the green–yellow region (500–600 nm) of differential reflectance curves. In addition, a trough at ~684 nm and a peak at ~695 nm were visible. Irradiation with high blue light for 1 h induces quenching of chlorophyll fluorescence (Pfündel *et al.*, 2018). Thus, we may expect that the maxima of the chlorophyll emission spectrum (measured in intact leaves) corresponded to troughs in the high- minus low-light differential curves. Accordingly, the trough at ~684 nm is near a small peak at ~685 nm visible in the fluorescence emission spectrum of several angiosperm species (Chappelle *et al.*, 1985; Agati, 1998). The peak at 695 nm in the differential reflectance spectra appears to correspond to the inflection point between the overlapping peaks in the chlorophyll emission spectrum. As the main peak of the emission spectrum from intact leaves is located at ~740 nm, fluorescence quenching probably contributes to the steep decrease in the differential reflectance spectrum visible between 700 nm and 745 nm.

### Monitoring chloroplast movements in *Arabidopsis thaliana* wild type and chloroplast movement mutants

**Fig. 2.**
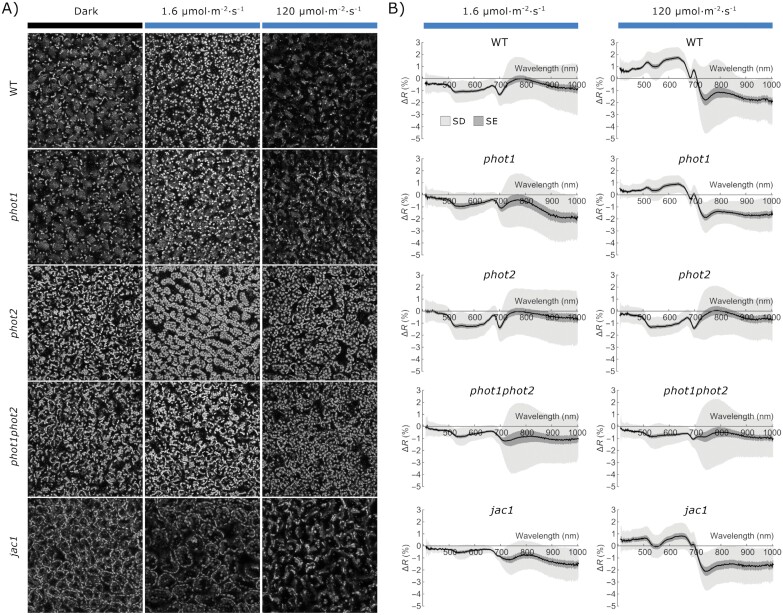
Chloroplast movements in *Arabidopsis thaliana* leaves. The blue light-induced movements were examined with (A) optical microscopy and (B) leaf reflectance spectra in *A. thaliana* rosette leaves of wild-type (WT), *phot1*, *phot2*, *phot1phot2*, and *jac1* mutant plants. (A) Chloroplast arrangements in palisade cells of detached leaves from 4-week-old dark-adapted plants were either kept in darkness (mock irradiation) or irradiated for 1 h with blue light of 1.6 µmol m^−2^ s^−1^ or 120 µmol m^−2^ s^−1^. Chloroplast arrangements were imaged with a laser-scanning confocal microscope, using chlorophyll autofluorescence. Maximum intensity projections were calculated from *Z*-stacks, recorded for ~90 μm, starting from the leaf’s upper surface. (B) Effect of blue light irradiation on the reflectance spectrum of the adaxial surface of Arabidopsis WT and phototropin mutant leaves. Detached leaves were irradiated with either 1.6 µmol m^−2^ s^−1^ or 120 µmol m^−2^ s^−1^ blue light for 1 h, with half of the blade covered with aluminum foil. The mean difference in reflectance spectra between irradiated and darkened halves was calculated from hyperspectral images. The sample size is 30 leaves for each combination of the plant line and irradiation conditions. The SD is shown in light gray, and the SE is in dark gray.

To better understand the relationship between chloroplast positioning and leaf reflectance, we examined light-induced changes in reflectance spectra of several *A. thaliana* mutants with disrupted chloroplast movements. The use of mutants was necessary to distinguish between spectral changes due to chloroplast movements and other processes of blue light-induced responses. In our sample, the mean total chlorophyll content was lower in the *phot1phot2* mutant than in the Arabidopsis wild type, though the difference was not statistically significant (Supplementary Fig. S2C). Microscopic observations (Fig. 2A) were performed to enable direct comparisons of the movement aberrations characteristic of mutants. The recorded spectra for Arabidopsis wild type and all chloroplast movement mutants are shown in Supplementary Fig. S4, S5. For Arabidopsis wild-type plants, patterns of differential leaf reflectance curves were similar to those in *N*. *benthamiana* (compare Fig. 1D, E and Fig. 2B). A decrease in the green–yellow region (500–600 nm) after blue light of 1.6 μmol m^–2^ s^–1^ and a saddle with a peak at ~695 nm after blue light of 120 μmol m^–2^ s^–1^ were observed. Spectra acquired for the *phot1* mutant resembled those of the wild type, as this mutant differs from the wild type only at very low light irradiance values, not investigated in this study. The *phot2* mutant exhibits chloroplast accumulation regardless of the light irradiance used, thus the differential leaf reflectance curves were similar for both irradiance values and resembled those of the wild type after blue light treatment of 1.6 μmol m^–2^ s^–1^. Curves that were representative of the *phot1phot2* mutant did not show specific differences for leaves irradiated with 1.6 μmol m^–2^ s^–1^ and 120 μmol m^–2^ s^–1^. The *jac1* mutant, which is characterized by chloroplast avoidance at both investigated irradiance values, exhibited curves showing characteristic features for chloroplast avoidance. It is worth noting that the dark positioning in the *jac1* mutant was distinct from that in the wild type and *phot1*, which differed from that in the *phot2* and *phot1phot2* mutants.

### Performance of machine learning procedures for classification of leaves according to the position of chloroplasts

**Fig. 3.**
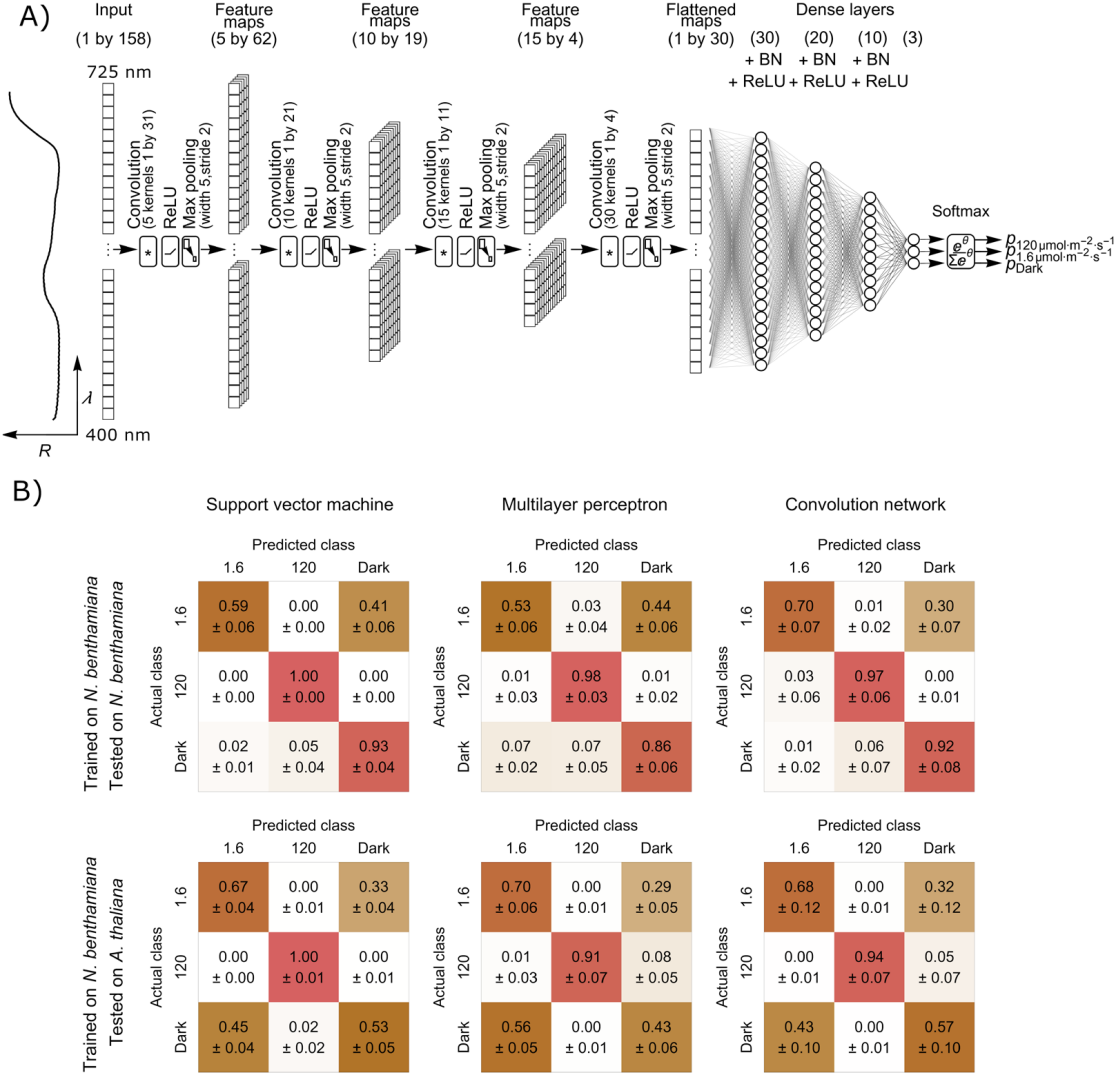
Machine learning approach to detection of chloroplast movements. (A) The architecture of the convolutional neuron network for classification of leaf reflectance spectra in the range 400–750 nm according to the chloroplast arrangement. The network consists of the initial convolutional part, four densely connected layers, and a softmax layer. The convolutional part consisted of four blocks, each with a 1-D convolution layer of variable kernel size and count, followed by a rectified linear unit (ReLU) and a maximum pooling layer, with a kernel size of five and stride of two. Each of the first three densely connected layers was followed by a batch normalization (BN) and a ReLU layer. The final softmax layer yields estimated probabilities of the input falling into each chloroplast arrangement class. (B) The confusion matrices showing the mean performance of the support vector machine with a linear kernel, multilayer perceptron, and convolutional network, trained on spectra recorded on *Nicotiana benthamiana* leaves and used to classify spectra recorded for the same species (upper row) or *Arabidopsis thaliana* (bottom row). Training was performed 20 times, and the standard deviation is shown.

Our next goal was to automatically assess chloroplast positioning using classification algorithms on the spectra extracted from hyperspectral images. Two types of neural networks were considered: a multilayer perceptron and a convolutional neural network (Malek *et al.*, 2018). The multilayer perceptron architecture was selected based on the validation set performance consisting of three layers, with 21, 10, and three neurons. The structure of the convolutional neural network, selected based on the cross-validation results for multiple trial architectures, is shown in Fig. 3A. The network consisted of four convolutional layers, interspersed with maximum pooling layers and ReLU activation functions. Feature maps generated by the last convolutional layer were concatenated into a single array that acted as an input for densely connected parts of the network, composed of four layers.

All three machine learning methods exhibited very high (91–100%) accuracy in distinguishing between spectra recorded on leaves with the chloroplast avoidance positioning and other types of chloroplast positioning (Fig. 3B). High accuracy was observed both when the trained and test sets came from the same species (*N. benthamiana*) and in the heterologous set up, in which the spectra recorded for *A. thaliana* were used as the test set. The performance of the tested procedures in distinguishing between spectra recorded on leaves exhibiting accumulation and dark positioning was substantially worse than that observed for the avoidance positioning. The best results were obtained using the convolutional network. For the *N. benthamiana* test dataset, 70% of spectra recorded on dark-adapted leaf parts and 92% of spectra recorded on leaves exhibiting the accumulation response were correctly classified.

### Chloroplast movement index

To identify wavelengths that can be used to formulate a vegetation index that could be used to distinguish leaves with different chloroplast arrangements, we employed the approach of the correlation matrix, proposed in Thenkabail *et al.* (2000). We used the formula of the normalized difference type


$$\begin{array}{l} ND\left(\lambda_{1},\lambda_{2}\right)=\frac{\;R_{\lambda 1}-R_{\lambda 2\;}}{R_{\lambda 1}+R_{\lambda 2}} \end{array}$$

**Fig. 4.**
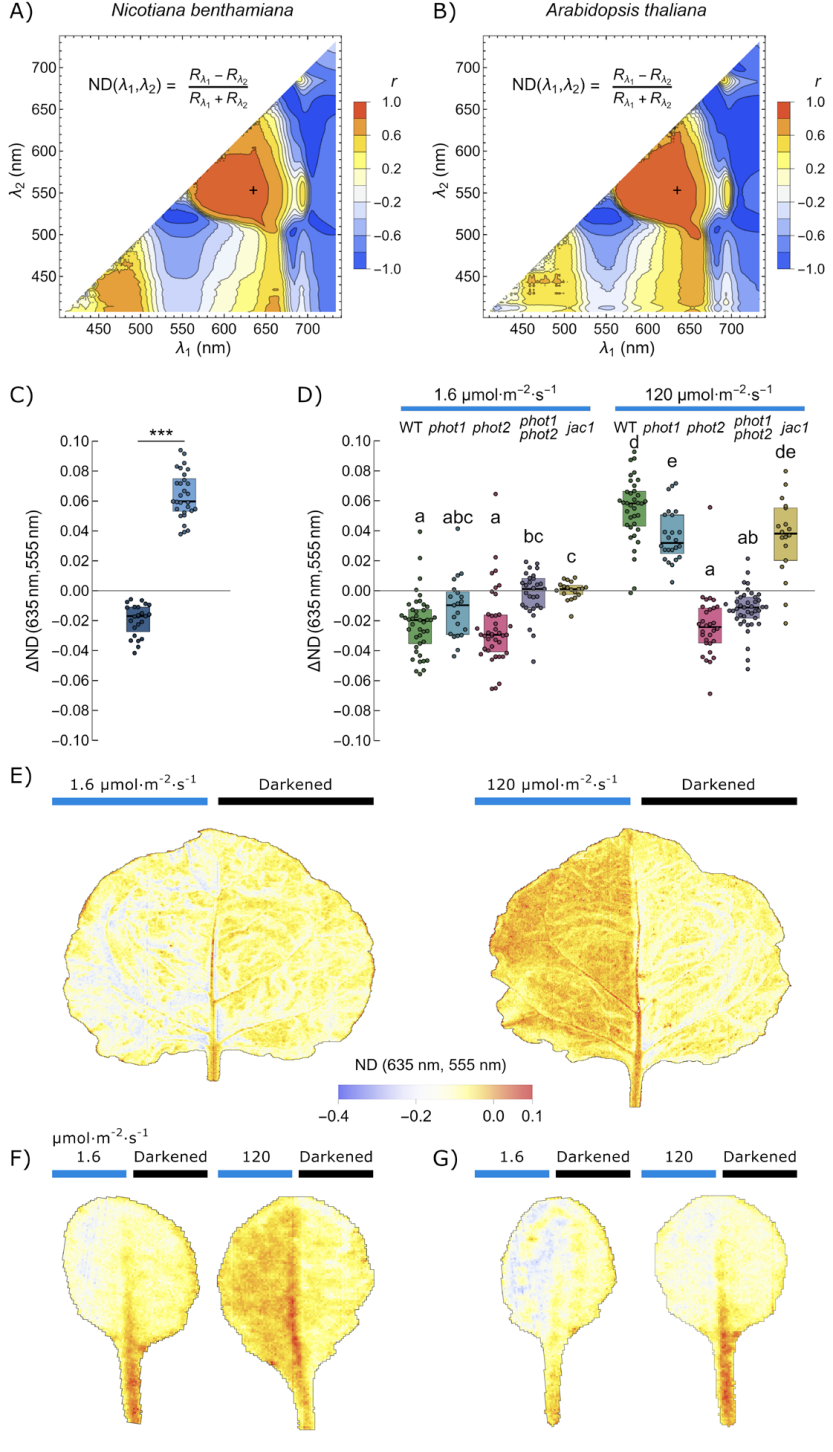
Classification of chloroplast positioning using a vegetation index of a normalized difference type. (A and B) Contour plots showing the biserial correlation between chloroplast positioning and the narrow band vegetation index of the general form specified in the plot, calculated from the leaf reflectance spectra for every combination of wavelengths λ_1_ (*x*-axis) and λ_2_ (*y*-axis) in the range of 400–750 nm. Spectra recorded for (A) *Nicotiana benthamiana* and (B) *Arabidopsis thaliana* leaves were used. Chloroplast accumulation was induced with 1.6 µmol m^−2^ s^−1^, and avoidance with 120 µmol m^−2^ s^−1^ of blue light. Blue and red areas correspond to indices that assume high values for leaves exhibiting accumulation and avoidance response, respectively. The pair of wavelengths (635 nm and 555 nm), used to calculate the ND index for chloroplast movements (CMI), positively correlated with the avoidance response and was marked with a cross. (C) The difference in the CMI between the dark-adapted and either low- or high-light irradiated *N. benthamiana* leaf halves. Each dot corresponds to one leaf. Statistical analysis was performed with the Welch *t*-test. (D) The difference in CMI in the dark-adapted and either low- or high-light-irradiated leaf halves of *A. thaliana* and chloroplast movement mutants (WT, green; *phot1*, blue; *phot2*, pink; *phot1phot2*, purple; *jac1*, yellow). Boxes that do not share any letter represent groups for which the means of transformed values differ at the 0.05 level (Tukey’s test, adjusted for multiple comparisons). (E–G) The CMI calculated for each pixel of hyperspectral images of (E) *N. benthamiana,* (F) *A. thaliana* wild type, and (G) the *phot2* mutant. In each leaf, one half was darkened, while the other was irradiated with either 1.6 µmol m^−2^ s^−1^ or 120 µmol m^−2^ s^−1^ of blue light.


Where *R*_λ1_ and *R*_λ2_ are relative reflectance values in narrow bands centered at two wavelengths λ_1_ and λ_2_ in the range of 400–750 nm (Fig. 4). For every pair of λ_1_ and λ_2_, the values of the index ND(λ_1_,λ_2_) from raw spectra and its biserial correlation with the chloroplast positioning (binomial variable with two levels) were calculated. The plots were similar for both investigated species, *N. benthamiana* (Fig. 4A) and *A. thaliana* (Fig. 4B). We chose the region between 550 nm and 670 nm, in which correlation was high, and identified the local maxima. We additionally took into account that illumination-induced changes in leaf reflectance in the range of 500–550 nm are likely to be partly due to changes in chloroplast thylakoid pH gradient and the de-epoxidation of violaxanthin to zeaxanthin (Gamon *et al.*, 1990), thus indices using wavelengths in this spectral region may be biased. Our final formula for the CMI, using the wavelength marked with a cross in Fig. 4A and B, is:

$$\begin{array}{l} CMI=\frac{\;R_{635}-R_{555\;}}{R_{635}+R_{555}} \end{array}$$

We calculated the values of this index for *Nicotiana* (Supplementary Fig. S6A) and Arabidopsis (Supplementary Fig. S6B) spectra and analyzed its performance to distinguish chloroplast accumulation from avoidance. The index took negative values for both species in the range of –0.1 to –0.45. The CMI proved to be very effective for *Nicotiana* leaves (Fig. 4C) irradiated with low and high blue light. For Arabidopsis wild type and chloroplast movement mutants (Fig. 4D), this index seemed to be able to distinguish between chloroplasts in the accumulation and avoidance positions depending on light irradiation regimes, but also the type of mutants. Wild-type plants, *phot1* and *phot2* mutants in low light and the *phot2* mutant in high light showed chloroplast accumulation. The *phot1phot2* mutant exhibited differences not dependent on chloroplast movements. Wild-type plants, *phot1*, and *jac1* mutants in high light demonstrated chloroplast avoidance. We also performed pixel-wise calculations of our CMI for whole leaves (Fig. 4E–G). Venation and petioles gave high values to the index, thus pre-processing of data may be necessary to exclude such areas before analysis, using for example NDVI. In lower resolution remote sensing, spectral unmixing may potentially be used to exclude the contribution of light reflected from tissues other than mesophyll.

### The impact of chloroplast movements on commonly used vegetation indices

**Fig. 5.**
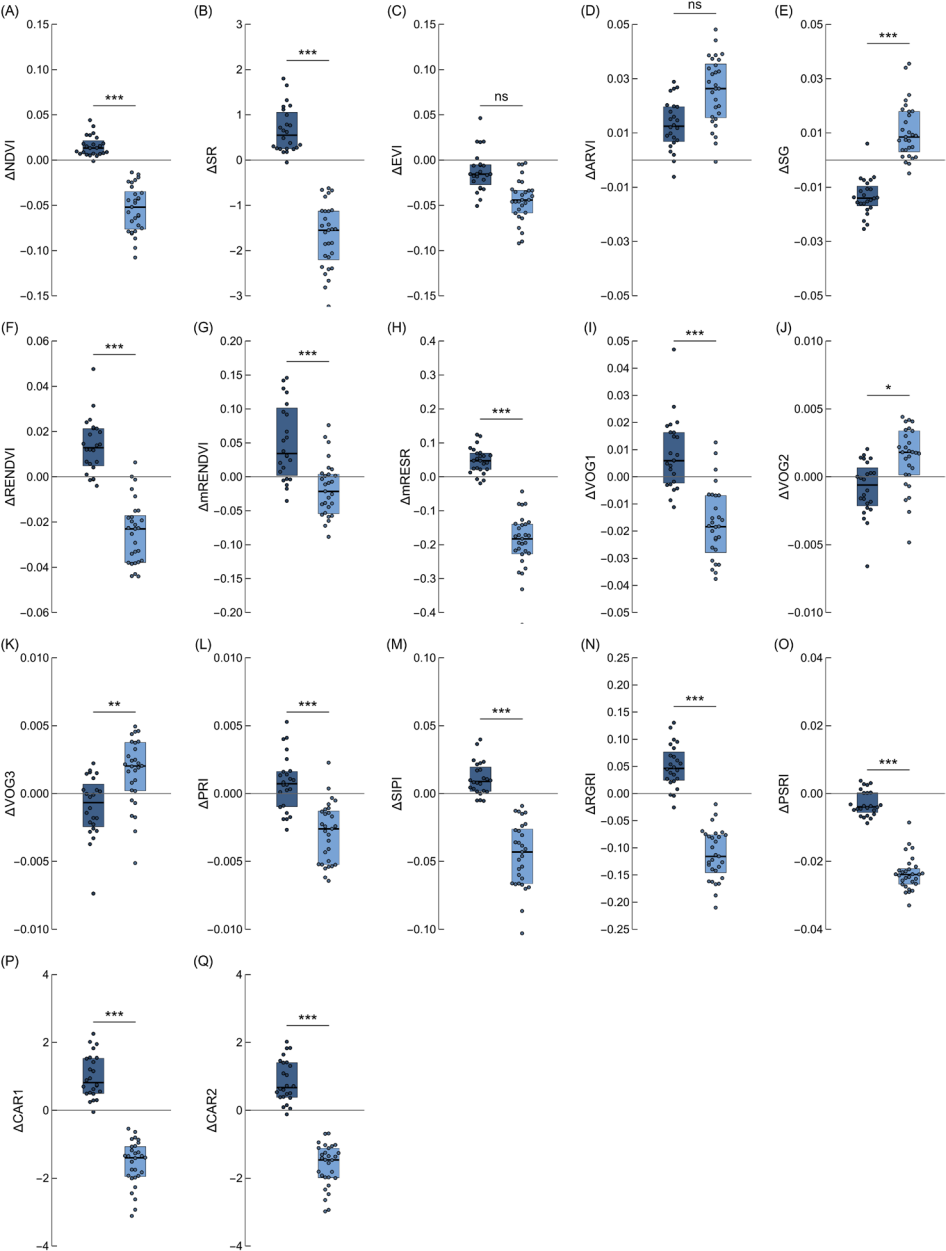
The difference between values of vegetation indices calculated from the spectra from darkened and irradiated parts of *Nicotiana benthamiana* leaves. Each dot corresponds to one leaf. Irradiation with low blue light of 1.6 µmol m^−2^ s^−1^ was used to induce chloroplast accumulation (dark blue boxes), with high blue light of 120 µmol m^−2^ s^−1^ to trigger the avoidance response (light blue boxes). The index formulae are given in Supplementary Table S1. The statistical significance of differences between means was examined using the Welch *t-*test.

We also calculated how chloroplast positioning affects vegetation indices calculated for *N. benthamiana* leaves. We used a set of indices that are sensitive to pigments and cell structure, as summarized in Behmann *et al.* (2014) (Supplementary Table S1). Chloroplast positioning after high light treatment reduced the values of several indices, especially NDVI (Fig. 5A; Supplementary Fig. S7A), SR (Fig. 5B; Supplementary Fig. S7B), RENDVI (Fig. 5F; Supplementary Fig. S7F), mRENDEVI (Fig. 5G; Supplementary Fig. S7G), mRESR (Fig. 5H; Supplementary Fig. S7H), VOG1 (Fig. 5I; Supplementary Fig. S7I), PRI (Fig. 5L; Supplementary Fig. S7L), SIPI (Fig. 5M; Supplementary Fig. S7M), RGRI (Fig. 5N; Supplementary Fig. S7N), PSRI (Fig. 5O; Supplementary Fig. S7O), CAR1 (Fig. 5P; Supplementary Fig. S7P), and CAR2 (Fig. 5Q; Supplementary Fig. S7Q). Chloroplasts in the avoidance position elevated SG (Fig. 5E; Supplementary Fig. S7E), VOG2 (Fig. 5J; Supplementary Fig. S7J), and VOG3 (Fig. 5K; Supplementary Fig. S7K) values. Chloroplast accumulation affected vegetation indices to a lesser extent, with diminished SG values and enhanced SR, RENDEVI, mRENDEVI, RGRI, CAR1, and CAR2. EVI (Fig. 5C; Supplementary Fig. S7C) and ARVI (Fig. 5D, Supplementary Fig. S7D) were not dependent on the position of chloroplasts.

**Fig. 6.**
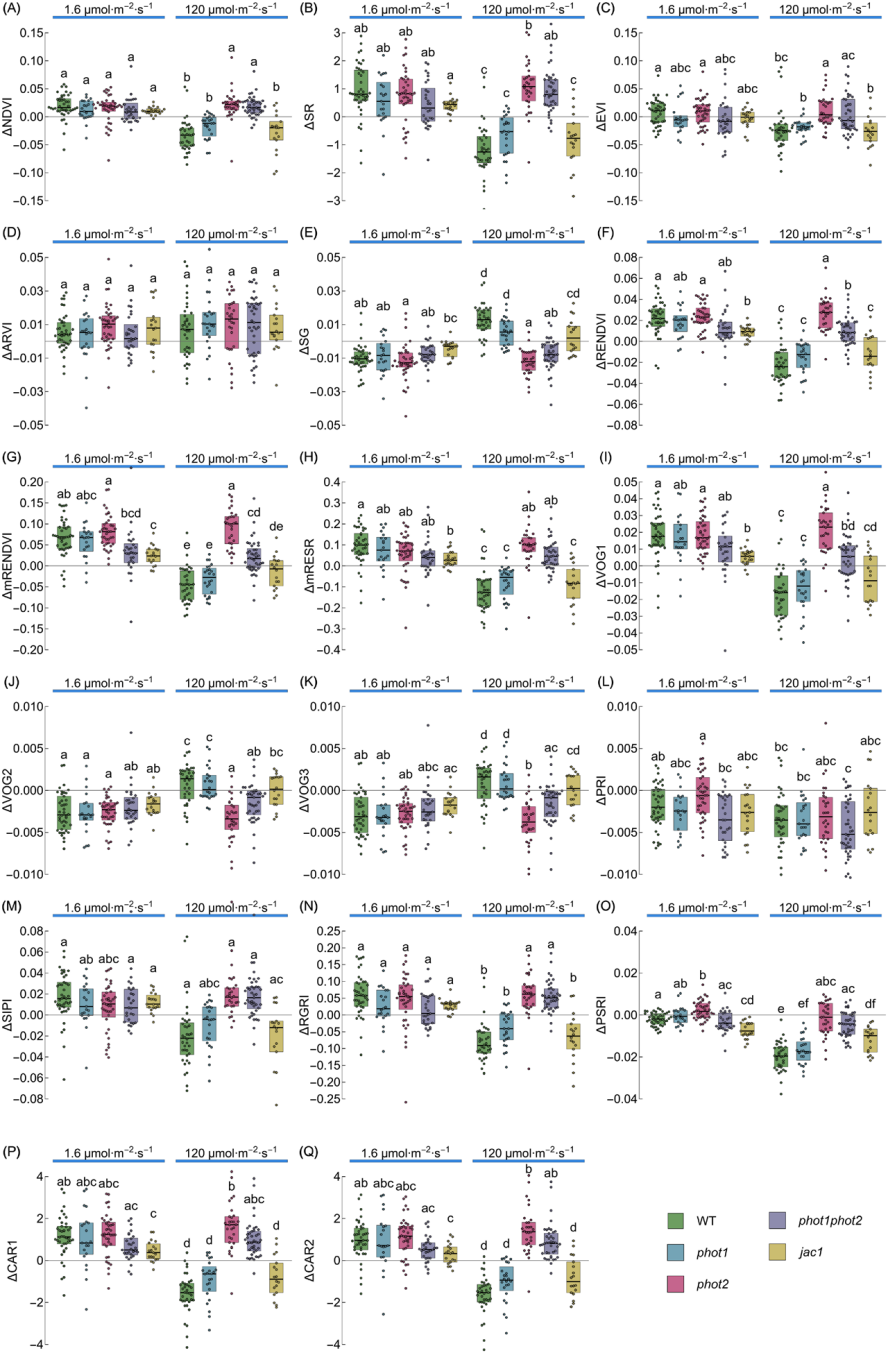
The difference between values of vegetation indices calculated from the spectra from dark-adapted and irradiated parts of *Arabidopsis thaliana* wild type and chloroplast movement mutants (WT, green; *phot1*, blue; *phot2*, pink; *phot1phot2*, purple; *jac1*, yellow). Each dot corresponds to one leaf. The irradiance to induce chloroplast accumulation was 1.6 µmol m^−2^ s^−1^ and to trigger chloroplast avoidance was 120 µmol m^−2^ s^−1^ of blue light. The index formulae are given in Supplementary Table S1. Boxes that do not share any letter represent groups for which the means of transformed values differ at the 0.05 level (Tukey’s test, adjusted for multiple comparisons).

In wild-type Arabidopsis, the direction of change (either elevation or reduction of the index value) was consistent with the results obtained for *Nicotiana* for all investigated indices apart from EVI and PRI (Supplementary Figs S7–S9; Figs 5, 6). The use of Arabidopsis chloroplast movement mutants enabled us to determine if the observed changes in the vegetative indices are related to differences in chloroplast arrangements. Values of several indices decreased in leaves showing the avoidance positioning, including NDVI (Fig. 6A; Supplementary Fig. S8A), SR (Fig. 6B; Supplementary Fig. S8B), RENDEVI (Fig. 6F; Supplementary Fig. S8F), mRENDEVI (Fig. 6G; Supplementary Fig. S8G), mRESR (Fig. 6H; Supplementary Fig. S8H), VOG1 (Fig. 6I; Supplementary Fig. S9I), SIPI (Fig. 6M; Supplementary Fig. S9M), RGRI (Fig. 6N; Supplementary Fig. S9N), PSRI (Fig. 6O; Supplementary Fig. S9O), CAR1 (Fig. 6P; Supplementary Fig. S9P), and CAR2 (Fig. 6Q; Supplementary Fig. S9Q). Such a decrease was not observed in mutants deficient in chloroplast avoidance, namely *phot2* and *phot1phot2*. The change in the value of the index mimicked the impact of low light of 1.6 μmol m^–2^ s^–1^ in those mutants. For indices in which chloroplast avoidance led to an increase in their value, such as SG (Fig. 6E; Supplementary Fig. S8E), VOG2 (Fig. 6J; Supplementary Fig. S9J), and VOG3 (Fig. 6K; Supplementary Fig. S9K), in both mutants, *phot2* and *phot1phot2*, diminished values of those indices were observed, similarly to those observed under low light. RENDEVI, mRENDEVI, and VOG1 seemed to be sensitive to chloroplast accumulation. Changes in EVI (Fig. 6C; Supplementary Fig. S8C), ARVI (Fig. 6D; Supplementary Fig. S8D), and PRI (Fig. 6L; Supplementary Fig. S9L) did not correspond to chloroplast positioning in cells, as no differences were observed between the Arabidopsis wild type and chloroplast movement mutants.

## Discussion

### Assessment of chloroplast movements through analysis of hyperspectral images

Here we propose a new method of chloroplast movement assessment based on broad-spectrum reflectance imaging and extraction of information about chloroplast positioning contained in the reflectance spectrum, using either vegetation indices or machine learning procedures. This method appears to be more suited for high-throughput detection of chloroplast movements than methods employing hemispherical reflectance (Davis *et al.*, 2011; Baránková *et al.*, 2016), which requires direct contact of the measuring equipment with the leaf. In the work of Dutta *et al.* (2015), directional leaf reflectance of red light was used to visualize chloroplast movements in whole Arabidopsis rosettes. The use of white light as the measuring light allowed us to examine the effects of chloroplast positioning not only on the absolute reflectance but also on the shape of the spectrum and ratios between reflectance in selected bands, properties that are often more robust against artifacts or confounding factors (see, for example, Gamon and Surfus, 1999 in the context of pigment content estimation). These issues are relevant for field conditions when sunlight is used as measuring light. In this work, we have examined the effect of chloroplast movement on the reflectance of leaves for fixed irradiation and observation directions, close to the leaf normal. In general, reflectance is a function of wavelength, as well as the directions of light incidence and observation, a relationship described as a bidirectional reflectance function (Nicodemus, 1965). Complete assessment of the potential of remote sensing methods for the detection of chloroplast movements will require further studies of blue light-induced changes in the whole bidirectional reflectance function. An approach to mitigate the detrimental effects of specular reflection on the accuracy of chloroplast movement detection has been proposed in Hermanowicz *et al.* (2023, Preprint).

Averaged hyperspectral images of partly irradiated leaves show a clear difference in the magnitude of visible light reflectance between the irradiated and non-irradiated parts (Fig. 1F). A study on maize suggested that an increase in photosynthetically active radiation reflectance resulting from water stress might stem from the chloroplast avoidance movement (Zygielbaum *et al.*, 2012). Irradiation with blue light affects the shape of the reflectance spectrum (Fig. 1D, E). The measurements performed on Arabidopsis mutants with disrupted chloroplast movements indicate that the observed blue light-induced changes in the spectrum are to a large extent caused by chloroplast relocations (Fig. 2). This suggests that it might be possible to use the information contained in the reflectance spectrum to classify leaves according to the chloroplast positioning. We found that machine learning procedures, in particular the convolution network, exhibited good overall performance in the classification of reflectance spectra of leaves according to the chloroplast positioning (Fig. 3). The support vector machine and neural networks can reliably distinguish leaves exhibiting chloroplast avoidance positioning from the dark-adapted and low-light-treated leaves, even when data used for training and testing come from different species (*N. benthamiana* and *A. thaliana*). This indicates that it may be possible to detect chloroplast avoidance even in spectra recorded on heterogeneous vegetation patches.

The CMI proposed in this work is the first effort to establish vegetation indices relevant for monitoring chloroplast movements in field conditions (Fig. 4). The differences between values of the index for dark-adapted and irradiated leaves for both investigated species *A. thaliana* and *N. benthamiana* are substantial. Differences of smaller magnitude are observed between dark-adapted and low-light-irradiated leaves. The index correctly characterizes chloroplast positions in Arabidopsis mutants with disrupted chloroplast movements and positioning. This suggests that the index is sensitive to chloroplast movements and not to other blue light-dependent processes. As phototropin mutations affect the leaf morphology and the mesophyll architecture (Kozuka *et al.*, 2011), good performance of the index for the Arabidopsis mutants suggests that it may be relatively robust against changes in the leaf structure.

### The impact of chloroplast movements on commonly used vegetation indices

Our results indicate that chloroplast movements may affect several vegetation indices commonly used for remote sensing of physiological traits. To assess the practical significance of this phenomenon, it is necessary to compare the observed changes caused by chloroplast relocations with the magnitude of variation of the indices due to changes in the traits that are usually monitored with them. Analysis of beech forests indicates that vegetation indices are affected by the light conditions in which leaves develop. They generally perform well with sun-lit leaves, but poorly with shaded leaves (Sonobe and Wang, 2017). In this work, we show that chlorophyll-dependent indices, characterized by simple formulae, such as RENDVI, mRENDVI, mRESR, SR, as well as SG, are affected by chloroplast positioning. The values for RENDEVI or mRENDVI fall in the range of 0–0.7 for various leaf types (Sims and Gamon, 2002), and thus may be significantly influenced by the chloroplast relocations. This is especially prominent when differences between dark and lit leaf halves of the investigated Arabidopsis mutants are analyzed. mRESR values are between 0 and 4 for a variety of leaf types and species. Low values of mRESR, comparable with those calculated in this study, are observed for leaves that are tough or pubescent (e.g. from drought-deciduous species) and are characterized by high surface reflectance (Sims and Gamon, 2002). The RGRI representing the anthocyanin/chlorophyll ratio is also substantially affected by chloroplast movements, with ΔRGRI values of ~0.1. RGRI values measured for *Quercus agrifolia* depend on leaf age in the range of 0.5–1.5. SR values mainly fall into the range of 0–20 as observed for paddy rice fields in Italy and 10–20 for clonal *Populus* (Thenkabail *et al.*, 2018). By contrast, the influence of chloroplast positioning is negligible for chlorophyll-dependent indices characterized by more complex formulae, such as the EVI and ARVI. The values of EVI, ARVI, SIPI, and PRI seem not to depend on chloroplast positioning, as changes in their values did not correlate in Arabidopsis phototropin mutants (Fig. 6). SIPI values of ~1 are shared for a wide variety of plant species, except for evergreen species (Sims and Gamon, 2002). PRI values fall in the range of –0.1 to 0.1. except for winter deciduous and evergreen species (Sims and Gamon, 2002). The values of VOG1 and NDVI are very similar between groups for both *Nicotiana* and Arabidopsis wild type and chloroplast movement mutants. The observed differences due to chloroplast positioning are relatively small, although statistically significant. However, NDVI values representing active vegetation are in the range of 0.7–0.9, with a calculated uncertainty value of ~0.02 (Borgogno-Mondino *et al.*, 2016). The difference in NDVI between chloroplast dark and avoidance positions is ~0.05, which is significantly above the uncertainty levels for this index.

The accuracy of methods for pigment content estimation from vegetation reflectance spectra is usually lower for carotenoids than for chlorophylls (Hill *et al.*, 2019), both at the leaf (Gitelson *et al.*, 2006) and at the canopy (Asner *et al.*, 2015) levels. This lower accuracy reported for carotenoids is at least partly due to their lower content. However, carotenoid-dependent indices, CAR1 and CAR2, are substantially influenced by chloroplast relocations. For tree species (maple, chestnut, and beech), a ΔCAR2 of 1 corresponds to a difference in carotenoid content of 1.8 nmol cm^–2^ (0.018 mmol m^–2^). A similar, although non-linear, relationship between carotenoid content and ΔCAR1 is observed for maple leaves (Gitelson *et al.*, 2002). For a survey investigating leaves from a wide range of species representing different functional types, carotenoid content fell in the range of 0–0.5 mmol m^–2^ (Sims and Gamon, 2002). The PSRI, useful for monitoring stress and senescence, is also significantly influenced by chloroplasts in the avoidance position. The difference that stems from chloroplast positioning may affect PSRI readings, as ΔPSRI values are in the range of 0.02. Values between –0.05 and 0.05 are observed for several annual, deciduous, and evergreen species (Sims and Gamon, 2002).

### Conclusion

We present a contactless method of chloroplast movement detection through hyperspectral imaging. It serves as a proof of concept for a high-throughput, remote technique applicable to field conditions. We also propose a new vegetation index of a normalized difference type, useful for assessing the positions of chloroplasts and distinguishing between the accumulation and avoidance responses. Finally, we draw the attention of the community to the impact that chloroplast movement may exert on remote sensing studies based on leaf reflectance. Several commonly used vegetation indices might be affected by the chloroplast positioning in mesophyll cells.

## Supplementary data

The following supplementary data are available at *JXB* online.

Table S1. Formulae of vegetation indices calculated from the spectra obtained on *Arabidopsis thaliana* and *Nicotiana benthamiana.*

Fig. S1. The procedure used for extraction of relative reflectance spectra of leaves and estimation of noise levels.

Fig. S2. Leaf chlorophyll content analyzed in *Nicotiana benthamiana* and *Arabidopsis thaliana* leaves.

Fig. S3. Leaf reflectance spectra recorded for dark-adapted and illuminated leaf halves of *N. benthamiana.*

Fig. S4. Leaf reflectance spectra recorded for dark-adapted and illuminated leaf halves of *A thaliana* wild type and chloroplast movement mutants.

Fig. S5. Leaf reflectance spectra recorded for dark-adapted and illuminated leaf halves of *A. thaliana* wild type and chloroplast movement mutants.

Fig. S6. Chloroplast movement index calculated value for leaf halves of *Nicotiana benthamiana* or *Arabidopsis thaliana* wild type and chloroplast movement mutants either kept in darkness or irradiated.

Fig. S7. Vegetation indices calculated for leaf halves of *Nicotiana benthamiana* either kept in darkness or irradiated.

Fig. S8. Vegetation indices calculated for leaf halves of *Arabidopsis thaliana* and chloroplast movement mutants either kept in the dark or irradiated.

Fig. S9. Vegetation indices calculated for leaf halves of *Arabidopsis thaliana* and chloroplast movement mutants either kept in the dark or irradiated.

erae407_suppl_Supplementary_Table_S1_Figures_S1-S9

## Data Availability

The data that support the findings of this study are available in the supplementary data of this article. Raw data are available from the corresponding author upon reasonable request.

## Abbreviations

ARVI: atmospherically resistant vegetation index
CAR1: carotenoid reflectance index 1
CAR2: carotenoid reflectance index 2
CMI: chloroplast movement index
EVI: enhanced vegetation index
NDVI: normalized difference vegetation index
mRENDVI: modified red edge NDVI
mRESR: modified red edge simple ratio index
PRI: photochemical reflectance index
PSRI: plant senescence reflectance index
RENDVI: red edge NDVI
RGRI: red–green ratio index
SG: sum green index
SIPI: structure-insensitive pigment index
SR: simple ratio index
VOG1: Vogelmann red edge index 1
VOG2: Vogelmann red edge index 2
VOG3: Vogelmann red edge index 3.
