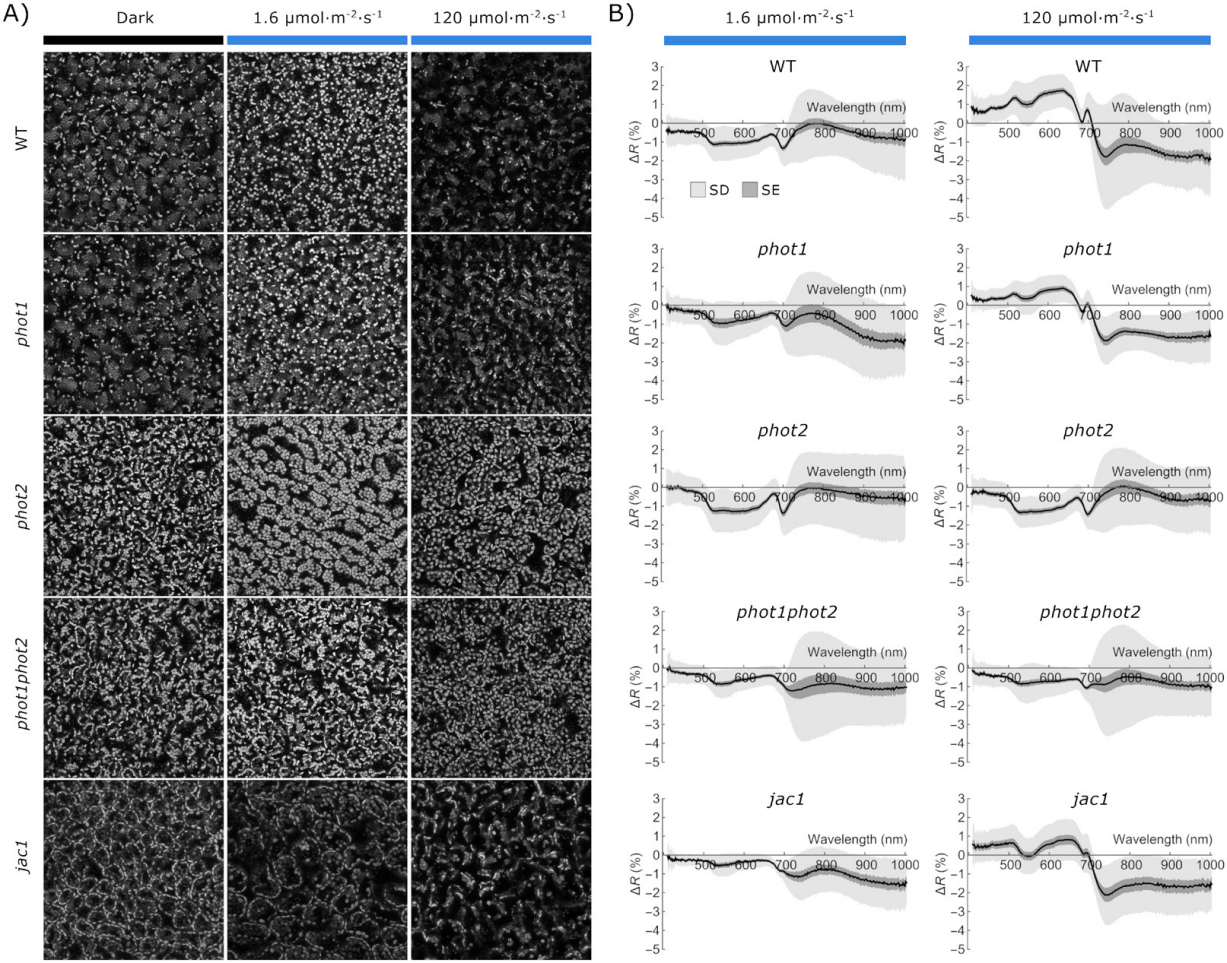# Correction to: Hyperspectral imaging for chloroplast movement detection

**DOI:** 10.1093/jxb/eraf043

**Published:** 2025-02-07

This is a correction to:

Paweł Hermanowicz, Justyna Łabuz, Hyperspectral imaging for chloroplast movement detection, *Journal of Experimental Botany*, 2024;, erae407, https://doi.org/10.1093/jxb/erae407

In the originally published version of this manuscript, charts for two mutants in Figure 2 were accidentally swapped during the proofing process.

This error has been corrected.