# Supplementary material for: Hyperspectral imaging for chloroplast movement detection
Source: J Exp Bot. 2024 Sep 27;76(3):882–98. doi: 10.1093/jxb/erae407 (PMC11805589; doi:10.1093/jxb/erae407)
Supplement: erae407_suppl_Supplementary_Table_S1_Figures_S1-S9 [file erae407_suppl_supplementary_table_s1_figures_s1-s9.pdf]

Hyperspectral imaging for chloroplast movement detection

Paweł Hermanowicz, Justyna Łabuz*

Supplementary Data

Table S1: Formulae of Vegetation Indices calculated from the spectra obtained on *Arabidopsis thaliana* and *Nicotiana benthamiana*

Normalized Difference Vegetation Index (NDVI) – Chlorophyll

$$\frac{R_{800} - R_{670}}{R_{800} + R_{670}}$$

Simple Ratio Index (SR) – Chlorophyll

$$\frac{R_{800}}{R_{670}}$$

Enhanced vegetation Index (EVI) – Chlorophyll

$$2.5\frac{R_{800} - R_{670}}{R_{800} + 6R_{670} - 7.5R_{490} + 1}$$

Atmospherically Resistant Vegetation Index (ARVI) – Chlorophyll

$$\frac{R_{800} - 2(R_{670} - R_{490})}{R_{800} + 2(R_{670} - R_{490})}$$

Sum Green Index (SG) – Greenness

$$\frac{1}{n}\sum_{i=500}^{599} R_i$$

Red Edge NDVI (RENDVI) – Chlorophyll

$$\frac{R_{750} - R_{705}}{R_{750} + R_{705}}$$

Modified Red Edge NDVI (mRENDVI) – Stress, Senescence

$$\frac{R_{750} - R_{705}}{R_{750} + R_{705} - 2R_{445}}$$

Modified Red Edge Simple Ratio Index (mRESR) – Chlorophyll

$$\frac{R_{750} - R_{445}}{R_{705} - R_{445}}$$

Vogelmann Red Edge Index 1 (VOG1) – Chlorophyll, Water

$$\frac{R_{740}}{R_{720}}$$

Vogelmann Red Edge Index 2 (VOG2) – Chlorophyll, Water

$$\frac{R_{734} - R_{747}}{R_{715} + R_{726}}$$

Vogelmann Red Edge Index 3 (VOG3) – Chlorophyll, Water

$$\frac{R_{734} - R_{747}}{R_{715} + R_{720}}$$

Photochemical Reflectance Index (PRI) – Photosynthesis, Carot.

$$\frac{R_{531} - R_{570}}{R_{531} + R_{570}}$$

Structure Insensitive Pigment Index (SIPI) – Carot.-Chl.-Ratio

$$\frac{R_{800} - R_{445}}{R_{800} + R_{680}}$$

Red Green Ratio Index (RGRI) – Anth.-Chl.-Ratio

$$\frac{Mean(R_{500-600})}{Mean(R_{600-700})}$$

Plant Senescence Reflectance Index (PSRI) – Stress, Senescence

$$\frac{R_{680} - R_{500}}{R_{750}}$$

Carotenoid Reflectance Index 1 (CAR1) – Carotenoids

$$\frac{1}{R_{510}} - \frac{1}{R_{550}}$$

Carotenoid Reflectance Index 2 (CAR2) – Carotenoids

$$\frac{1}{R_{510}} - \frac{1}{R_{700}}$$

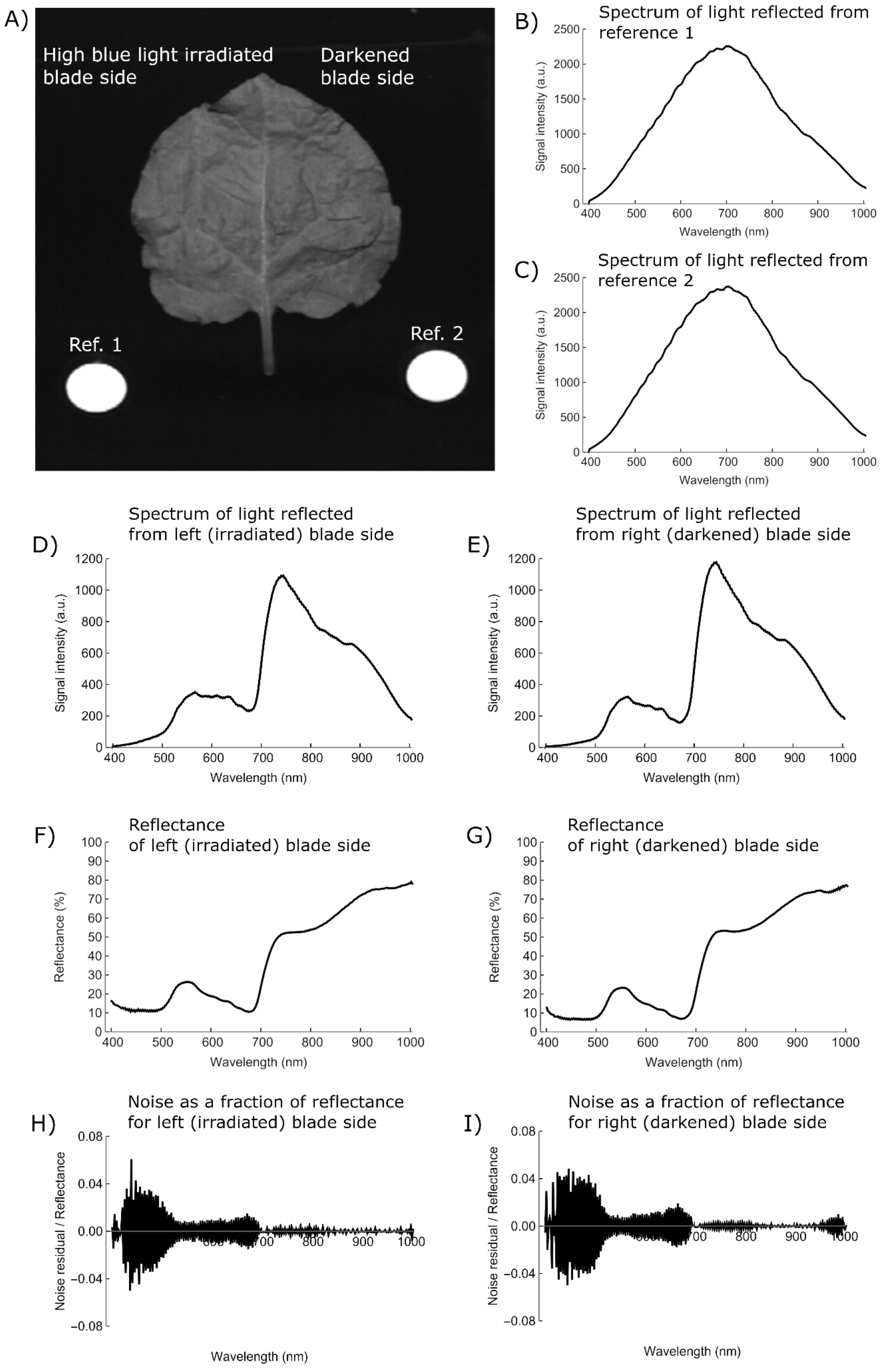
A)
High blue light irradiated blade side
Darkened blade side
Ref. 1
Ref. 2
B)
Spectrum of light reflected from reference 1
Signal intensity (a.u.)
Wavelength (nm)
C)
Spectrum of light reflected from reference 2
Signal intensity (a.u.)
Wavelength (nm)
D)
Spectrum of light reflected from left (irradiated) blade side
Signal intensity (a.u.)
Wavelength (nm)
E)
Spectrum of light reflected from right (darkened) blade side
Signal intensity (a.u.)
Wavelength (nm)
F)
Reflectance of left (irradiated) blade side
Reflectance (%)
Wavelength (nm)
G)
Reflectance of right (darkened) blade side
Reflectance (%)
Wavelength (nm)
H)
Noise as a fraction of reflectance for left (irradiated) blade side
Noise residual / Reflectance
Wavelength (nm)
I)
Noise as a fraction of reflectance for right (darkened) blade side
Noise residual / Reflectance
Wavelength (nm)

Fig. S1. The procedure used for extraction of relative reflectance spectra of leaves and estimation of noise levels. (A) One side of a detached *N. benthamiana* leaf was irradiated with blue light. The other side was covered with aluminum foil (darkened). White polytetrafluoroethylene (PTFE) disks served as references. (B, C) The average spectra of light reflected from reference disks, corresponding to the spectrum of the halogen lamp. (D, E) Raw average spectra of light reflected from the irradiated (D) and darkened (E) sides of the blade. (F, G) Reflectance of leaf blades. Spectra from PTFE disks were used as the 100% reference. (H, I) Estimation of the relative noise. The reflectance curves for irradiated and darkened blade sides were smoothed with the local regression method (tricube function used for weights, local polynomial degree of 2 was fitted using moving window spanning ca. 18 nm). The residual from the local regression served as a measure of noise. The noise was the greatest in the blue region, likely due to the relatively low output of halogen lamps in this range. The ratio of noise to reflectance reached 0.04 in the blue region.

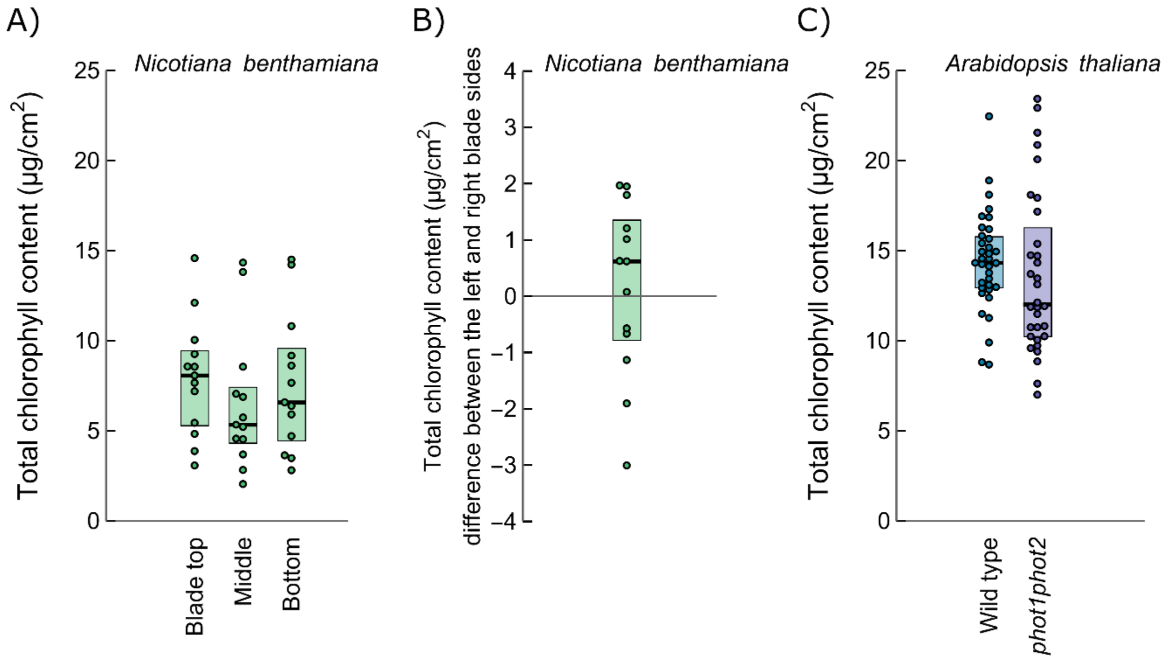

Fig. S2. Leaf chlorophyll content analyzed in *Nicotiana benthamiana* (A, B) and *Arabidopsis thaliana* leaves (C). For *N. benthamiana* leaves, chlorophyll content was assessed in the top, middle, and bottom parts of the blade of dark-adapted plants, separately on the left and right sides. The difference (B) in chlorophyll content between the right and left sides of the leaf was also calculated. (C) or *Arabidopsis*, chlorophyll content was examined in the wild type and the *phot1phot2* mutant.

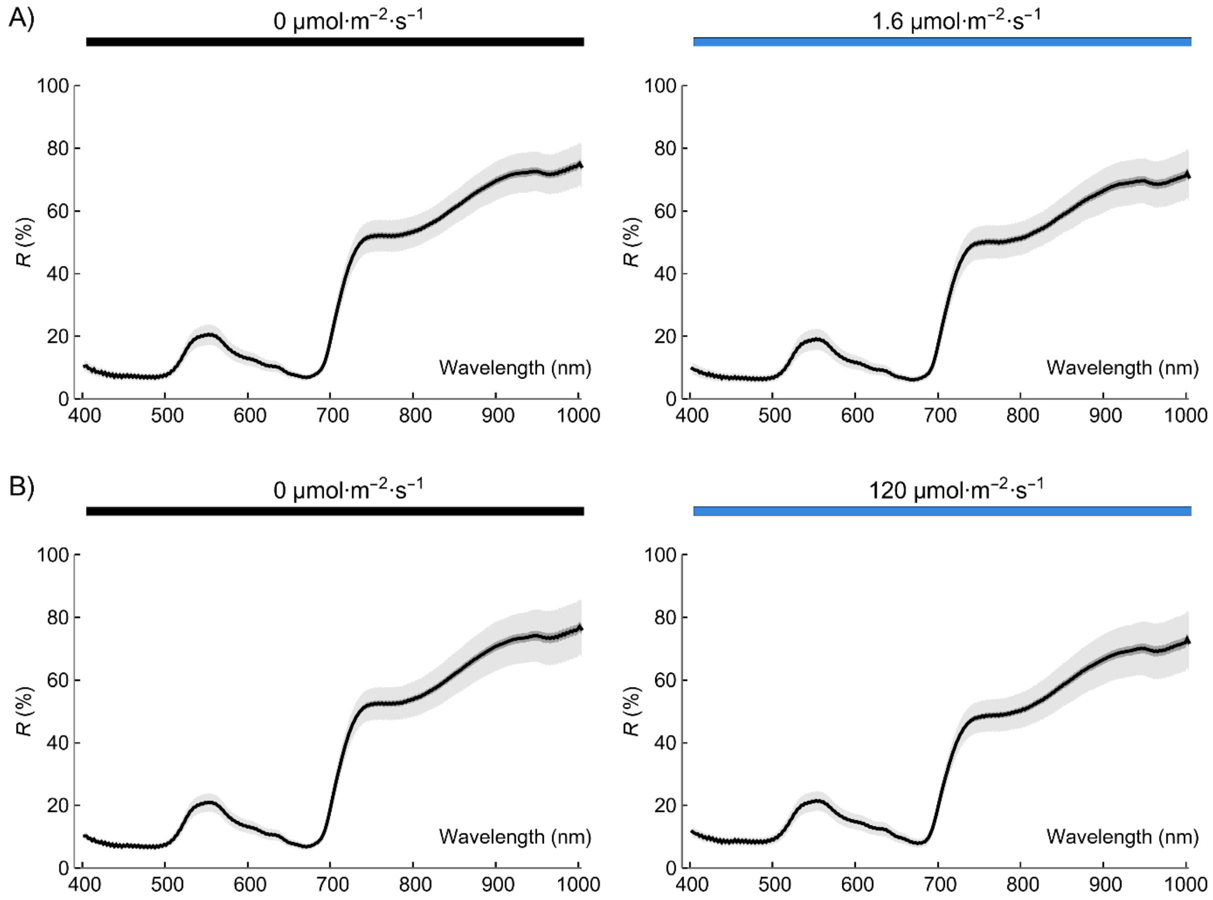

Fig. S3. Leaf reflectance spectra recorded for dark-adapted and illuminated leaf halves of *N. benthamiana*. Detached leaves were irradiated with either (A) 1.6 or (B) 120 mmol m^{-2} s^{-1} of continuous blue light (455 nm) for 1 h, with half of the blade covered with aluminum foil.

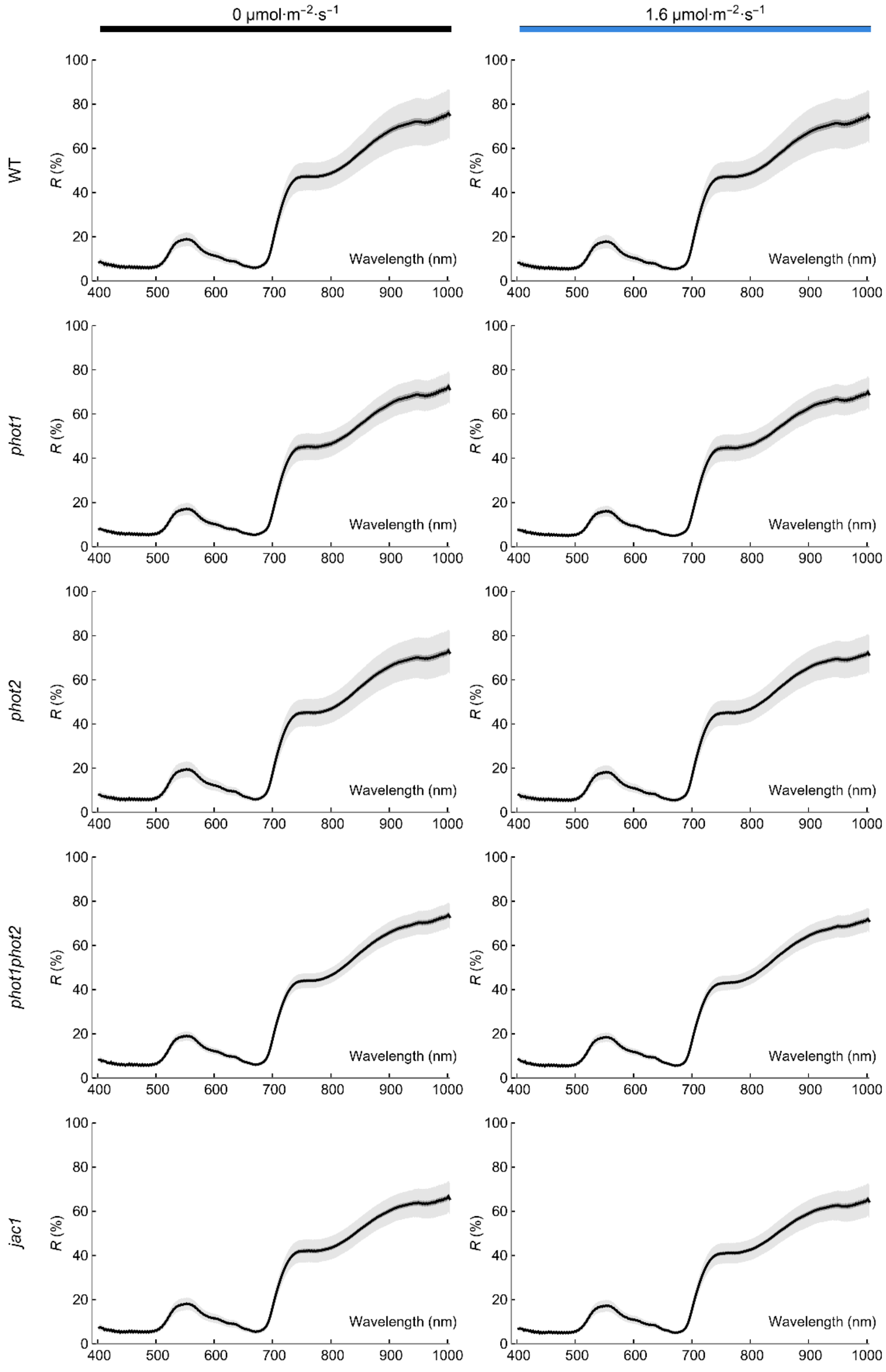

Fig. S4. Leaf reflectance spectra recorded for dark-adapted and illuminated leaf halves of *A thaliana* wild-type and chloroplast movement mutants. Detached leaves were irradiated with either 1.6 mmol m^{-2} s^{-1} of continuous blue light (455 nm) for 1 h, with half of the blade covered with aluminum foil.

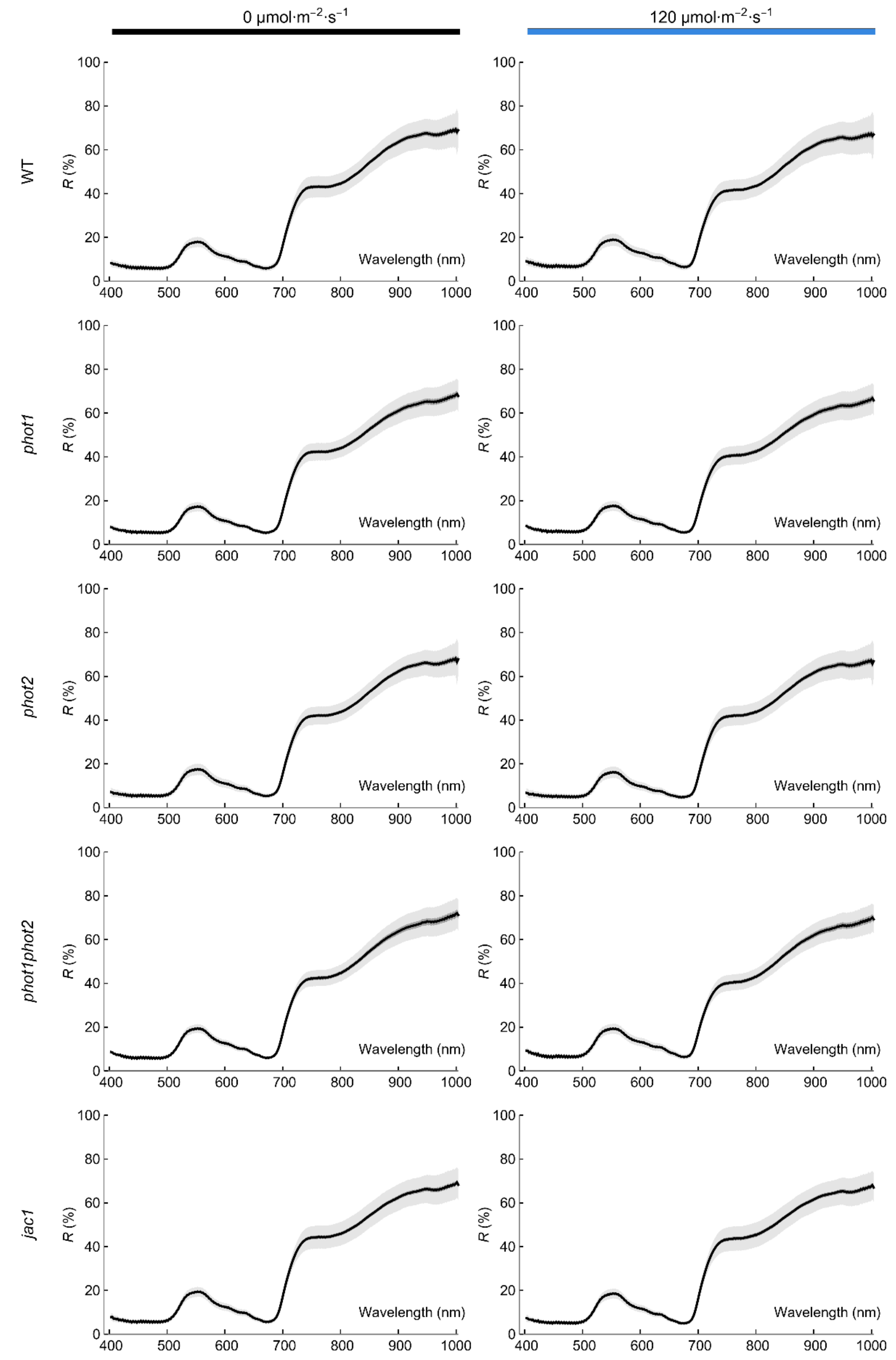

Fig. S5. Leaf reflectance spectra recorded for dark-adapted and illuminated leaf halves of *A thaliana* wild-type and chloroplast movement mutants. Detached leaves were irradiated with either 120 mmol m^{-2} s^{-1} of continuous blue light (455 nm) for 1 h, with half of the blade covered with aluminum foil.

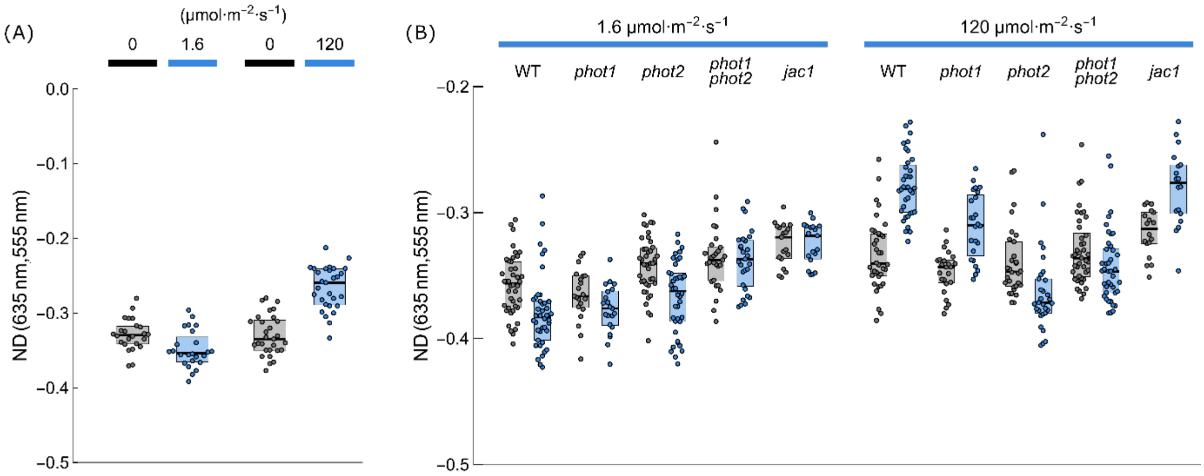

Fig. S6. Chloroplast Movement Index calculated value for leaf halves of (A) *Nicotiana benthamiana* or (B) *Arabidopsis thaliana* wild type and chloroplast movement mutants (*phot1*, *phot2*, *phot1phot2*, *jac1*) either kept in darkness (grey) or irradiated (blue). The irradiance to induce chloroplast accumulation was 1.6 $\mu mol \cdot m^{-2} \cdot s^{-1}$ and to trigger chloroplast avoidance was 120 $\mu mol \cdot m^{-2} \cdot s^{-1}$.

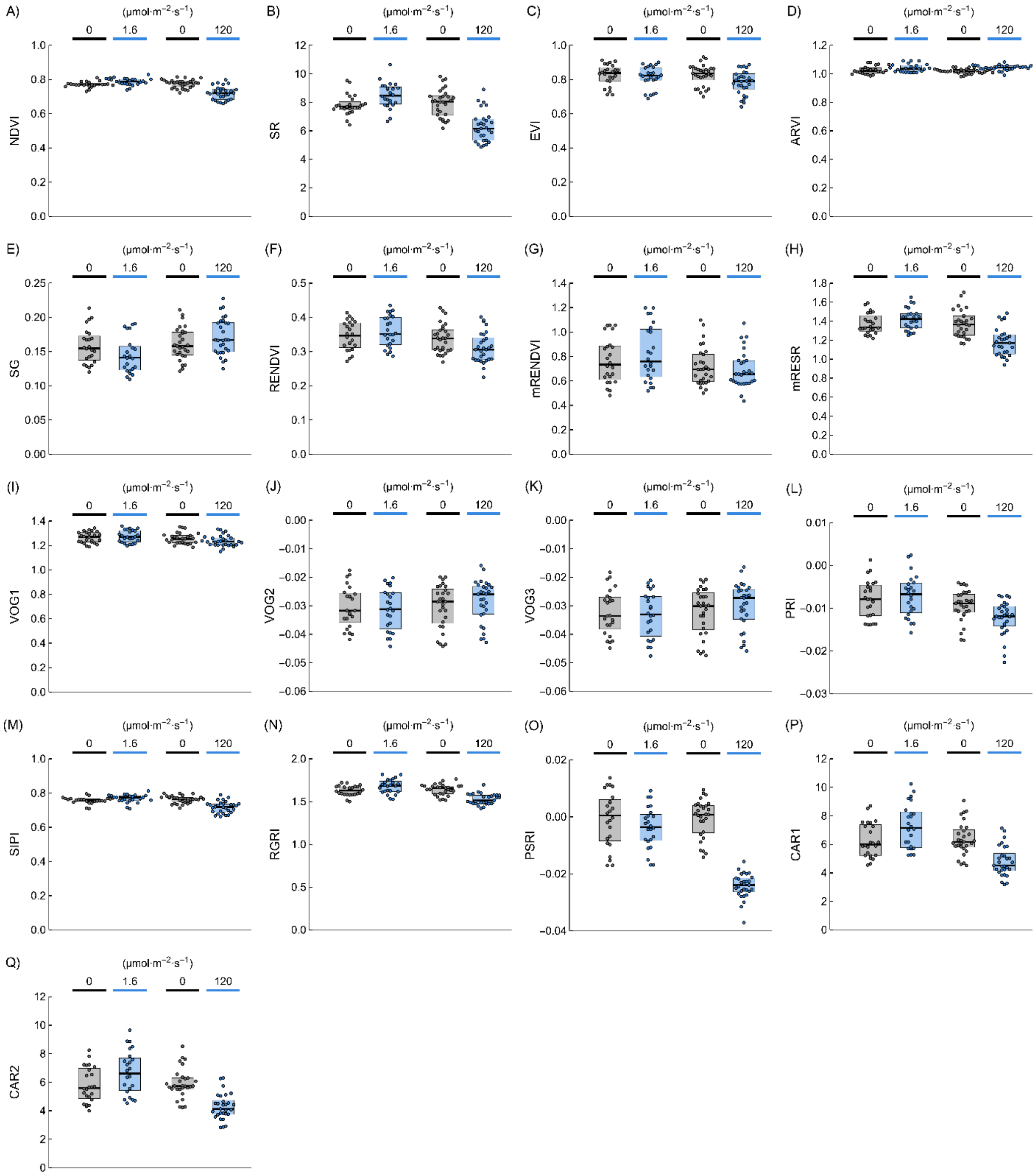

Fig. S7. Vegetation indices calculated for leaf halves of *Nicotiana benthamiana* either kept in the dark (grey) or irradiated (blue). The formulae for the indices are collected in the Supplementary Table S1. The irradiance to induce chloroplast accumulation was 1.6 μmol·m^{-2}·s^{-1} and to trigger chloroplast avoidance was 120 μmol·m^{-2}·s^{-1}. (A) NDVI, (B) SR, (C) EVI, (D) ARVI, (E) SG, (F) RENDVI, (G) mRENDEVI, (H) mRESR, (I) VOG1, (J) VOG2, (K) VOG3, (L) PRI, (M) SIPI, (N) RGRI, (O) PSRI, (P) CAR1, and (Q) CAR2.

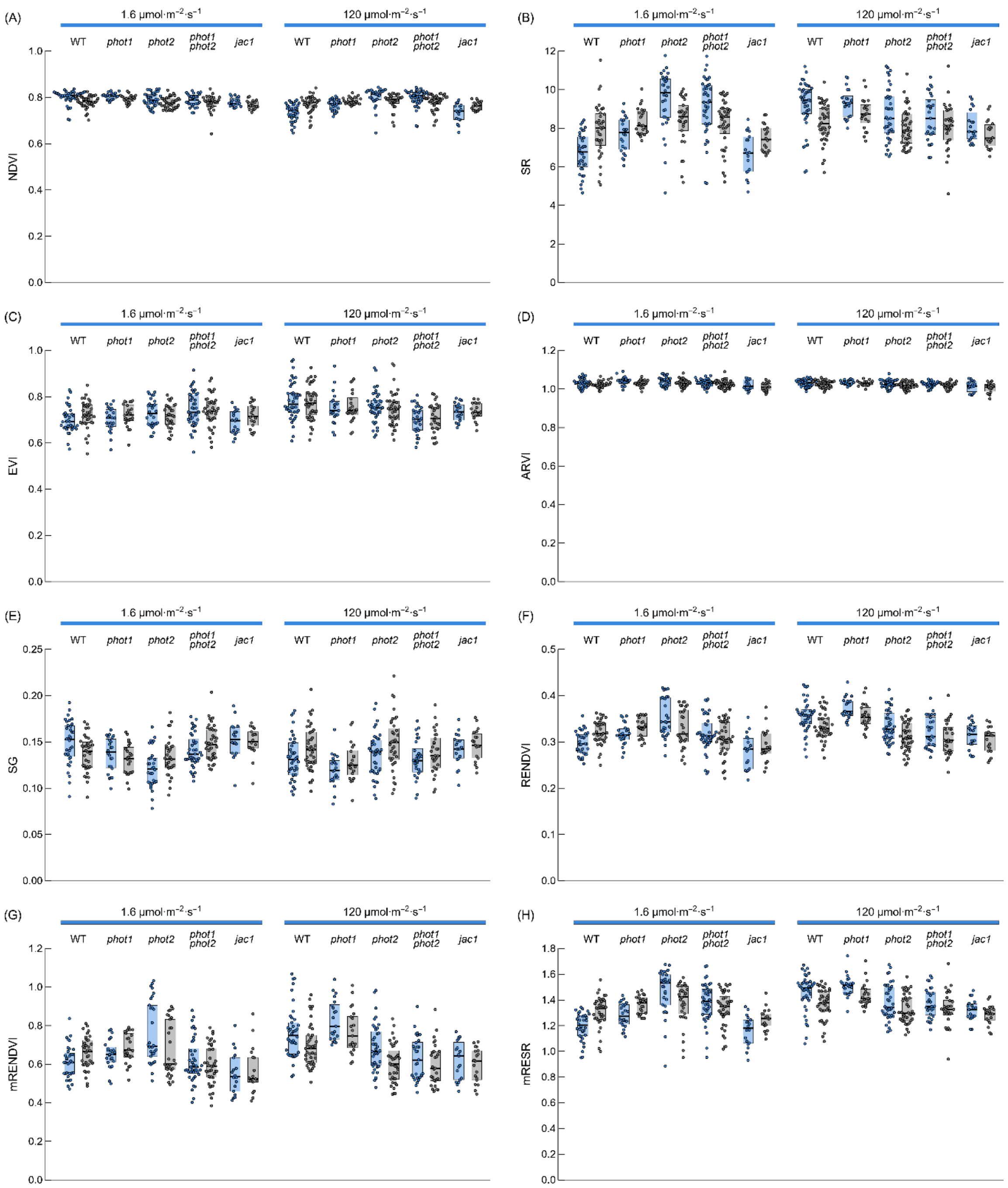

Fig. S8. Vegetation indices calculated for leaf halves of *Arabidopsis thaliana* and chloroplast movement mutants (*phot1*, *phot2*, *phot1phot2*, *jac1*) either kept in the dark (grey) or irradiated (blue). The formulae for the indices are collected in the Supplementary Table S1. The irradiance to induce chloroplast accumulation was 1.6 μmol·m^{-2}·s^{-1} and to trigger chloroplast avoidance was 120 μmol·m^{-2}·s^{-1}. (A) NDVI, (B) SR, (C) EVI, (D) ARVI, (E) SG, (F) RENDVI, (G) mRENDEVI, (H) mRESR.

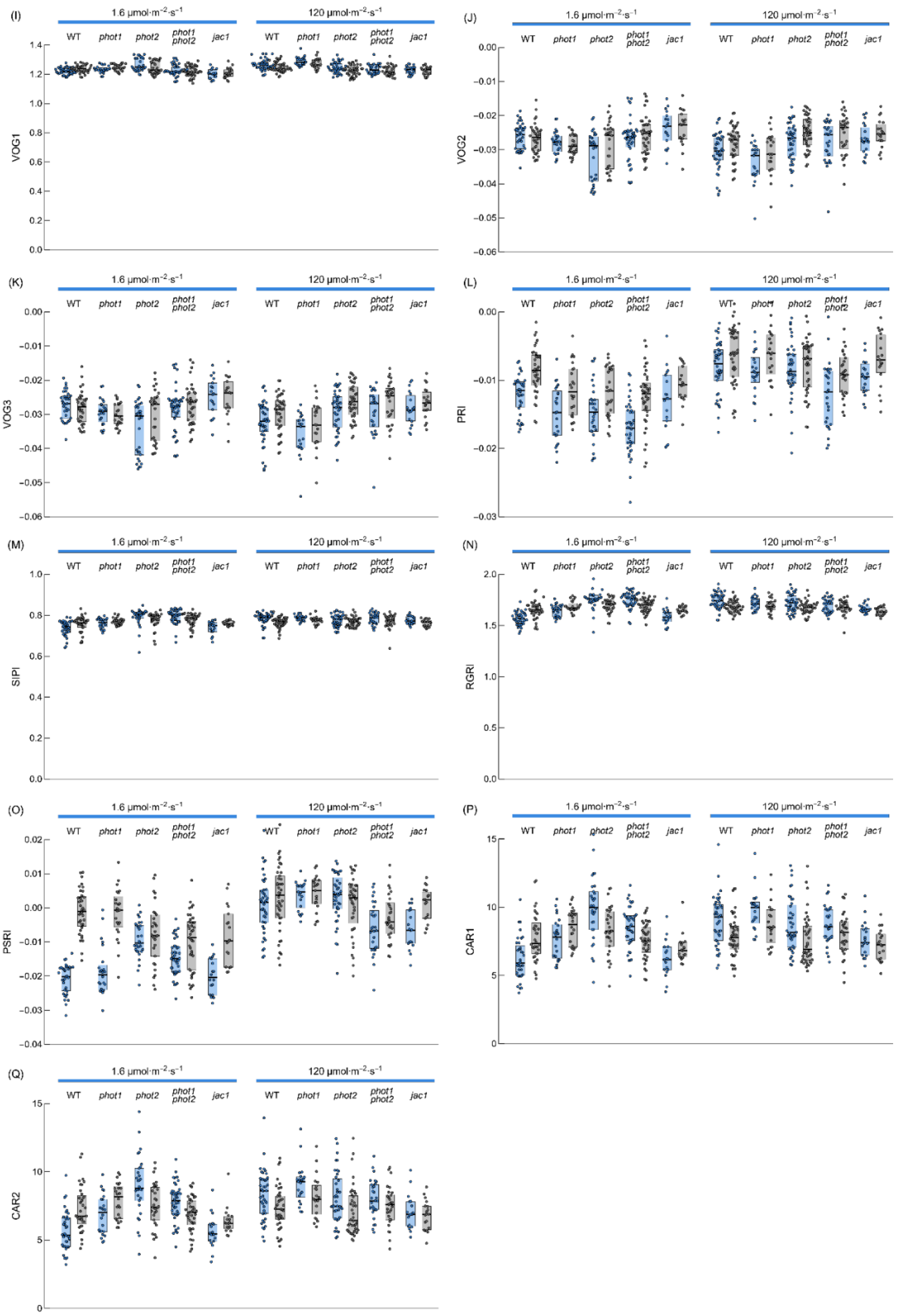

Fig. S9. Vegetation indices calculated for leaf halves of *Arabidopsis thaliana* and chloroplast movement mutants (*phot1*, *phot2*, *phot1phot2*, *jac1*) either kept in the dark (grey) or irradiated (blue). The formulae for the indices are collected in the Supplementary Table S1. The irradiance to induce chloroplast accumulation was 1.6 μmol·m^{-2}·s^{-1} and to trigger chloroplast avoidance was 120 μmol·m^{-2}·s^{-1}. (I) VOG1, (J) VOG2, (K) VOG3, (L) PRI, (M) SIPI, (N) RGRI, (O) PSRI, (P) CAR1, and (Q) CAR2.
